# The perinatal bereavement project: development and evaluation of supportive guidelines for families experiencing stillbirth and neonatal death in Southeast Brazil—a quasi-experimental before-and-after study

**DOI:** 10.1186/s12978-020-01040-4

**Published:** 2021-01-06

**Authors:** Heloisa de Oliveira Salgado, Carla Betina Andreucci, Ana Clara Rezende Gomes, João Paulo Souza

**Affiliations:** 1 Department of Social Medicine, Ribeirão Preto Medical School, University of São Paulo, Avenida dos Bandeirantes, 3900, Monte Alegre, Ribeirão Preto, SP 14049-900 Brazil; 2 Department of Medicine - Center for Biological and Health Sciences, Federal University of São Carlos, Rod. Washington Luiz, s/n, São Carlos, 13565-905 Brazil

**Keywords:** Stillbirth, Perinatal loss, Neonatal loss, Perinatal bereavement, Neonatal bereavement, Maternal grief, Bereavement protocol, Humanized birth assistance, Óbito fetal, Óbito perinatal, Óbito neonatal, Luto gestacional, Luto perinatal, Luto neonatal, Luto materno, Luto parental, Protocolo de luto perinatal, Humanização da assistência ao parto

## Abstract

**Background:**

For most parents, getting pregnant means having a child. Generally, the couple outlines plans and has expectations regarding the baby. When these plans are interrupted because of a perinatal loss, it turns out to be a traumatic experience for the family. Validating the grief of these losses has been a challenge to Brazilian society, which is evident considering the childbirth care offered to bereaved families in maternity wards. Positively assessed care that brings physical and emotional memories about the baby has a positive impact on the bereavement process that family undergoes. Therefore, this study aims to assess the effects supportive guidelines have on mental health. They were designed to assist grieving parents and their families while undergoing perinatal loss in public maternities in Ribeirão Preto, São Paulo state, Brazil.

**Method:**

A mixed-methods (qualitative/quantitative), quasi-experimental (before/after) study. The intervention is the implementation of bereavement supportive guidelines for women who experienced a stillbirth or a neonatal death. A total of forty women will be included. Twenty participants will be assessed before and twenty will be assessed after the implementation of the guidelines. A semi-structured questionnaire and three scales will be used to assess the effects of the guidelines. Health care professionals and managers of all childbirth facilities will be invited to participate in focus group. Data will be analyzed using statistical tests, as well as thematic analysis approach.

**Discussion:**

The Perinatal Bereavement guidelines are a local adaptation of the Canadian and British corresponding guidelines. These guidelines have been developed based on the families’ needs of baby memories during the bereavement process and include the following aspects: (1) Organization of care into periods, considering their respective needs along the process; (2) Creation of the Bereavement Professional figure in maternity wards; (3) Adequacy of the institutional environment; (4) Communication of the guidance; (5) Creation of baby memories. We expect that the current project generates additional evidence for improving the mental health of women and families that experience a perinatal loss.

*Trial registration* RBR-3cpthr

**Plain English summary:**

For many couples, getting pregnant does not only mean carrying a baby, but also having a child. Most of the time, the couple has already made many plans and has expectations towards the child. When these plans are interrupted because of a perinatal loss, it turns out to be a traumatic experience for the family.

In Brazilian culture, validating this traumatic grief is very difficult, especially when it happens too soon. The barriers can be noticed not only by the way society deals with the parents’ grief, but also when we see the care the grieving families receive from the health care establishment.

Creating physical and emotional memories might bring the parents satisfaction regarding the care they receive when a baby dies. These memories can be built when there is good communication throughout the care received; shared decisions; the chance to see and hold the baby, as well as collect memories; privacy and continuous care during the whole process, including when there is a new pregnancy, childbirth and postnatal period. With this in mind, among the most important factors are the training of health staff and other professionals, the preparation of the maternity ward to support bereaved families and the continuous support to the professionals involved in the bereavement.

This article proposes guidelines to support the families who are experiencing stillbirth and neonatal death. It may be followed by childbirth professionals (nurses, midwives, obstetricians and employees of a maternity ward), managers, researchers, policymakers or those interested in developing specific protocols for their maternity wards.

**Supplementary information:**

**Supplementary information** accompanies this paper at 10.1186/s12978-020-01040-4.

## RESUMO

**Contexto:**

Uma assistência avaliada positivamente por mães e pais que passaram pela perda perinatal permite a criação de memórias físicas e afetivas do bebê e possuem efeitos positivos no processo de luto da família. Este estudo avaliará os efeitos de uma diretriz de acolhimento na saúde mental de mulheres em processo de luto perinatal e neonatal em maternidades públicas do município de Ribeirão Preto (SP, Brasil).

**Método:**

Estudo de métodos mistos (quantitativo e qualitativo), quase-experimental (antes e depois). A intervenção é a implementação de diretrizes de acolhimento ao luto de mulheres que tiveram um natimorto ou óbito neonatal. Um total de quarenta mulheres serão incluídas. Vinte participantes serão avaliadas antes, e vinte após a implementação da diretriz de acolhimento nas instituições. Serão aplicadas três escalas e uma entrevista semiestruturada para avaliar os efeitos da diretriz. Profissionais da saúde e gestores serão convidados a participar de grupos focais. Os dados serão analisados por meio de testes estatísticos, e sob a metodologia de análise temática. A diretriz de acolhimento contará com material baseado em diretrizes canadense e britânica.

**Discussão:**

As diretrizes brasileiras de luto perinatal propostas são uma adaptação local das diretrizes canadense e britânica. Baseamo-nos na necessidade da família por memórias físicas e afetivas da criança morta para facilitar a vivência do processo do luto. Elas incluem os seguintes aspectos: (1) organização dos períodos da assistência a partir de suas respectivas necessidades, (2) criação do papel do Profissional do Luto, (3) ambientação das instituições, (4) disseminação das diretrizes e (5) criação de memórias do bebê. Espera-se que o projeto gere evidências adicionais para melhorar a saúde mental de mulheres e famílias que vivenciam uma perda perinatal.

## Background

The death of a baby during pregnancy, birth or postpartum is a traumatic experience to a woman and her family [1, 2]. However, it may be also a traumatic experience to the attending health care workers [2, 3]. Several factors make this a complex and marginalized issue, putting it at the bottom of the political agenda: stillbirths’ rates are not included in the Millennium Development Goals, nor tracked by the UN, nor in the Global Burden of Disease metrics [4], making it an invisible issue. Besides that, miscarriage, stillbirth and neonatal death lead to parental bereavement, resulting in a complex and eventually traumatic experience [5], a challenging issue to health professionals and to the family to cope. According to Parkes [6] “For most people in the Western world, the death of a child is the most tormenting and painful source of grief”.

Secondly, the belief the death of a child during pregnancy or after birth would be less difficult to deal with compared to the death of an older child is not true [1]. Actually, the main difference between these two situations is that society does not acknowledge perinatal losses; conversely, society minimizes it, makes it invisible and silences the experience parents are going through. This kind of grief is named disenfranchised grief, known as “(…) a loss that is not or cannot be openly acknowledged, publicly mourned, or socially supported” (Doka, 1989) [7]. Therefore, parents do not have their feelings of grief, sorrow, emptiness and helplessness socially validated [8]. Last but not least, parents, families and health care professionals do not expect pregnancy interruption, or a baby´s sudden death, which turns it in a more traumatic and difficult to cope situation [6].

It is estimated that a total of 2.6 million stillbirths took place globally in 2016 [9], and the estimates could be even higher considering underreporting of fetal death, and even higher if including perinatal deaths [3, 4]. The numbers by themselves make the issue a global priority. Nevertheless, they are just the tip of the iceberg.

A perinatal death impacts parents’ mental health and may trigger depressive symptoms, anxiety, post-traumatic stress disorders, suicidal ideation, panic and phobias [10]. It has also consequences in the social and economic spheres, with family crises, occupational difficulties and problems regarding high health care costs [3].

Women who had a stillborn baby may express guilt and question their competence for bearing a healthy baby. Besides, not only the grieving process may last for months and years, but it also impacts subsequent pregnancies [10, 11].

Given the importance of physical and mental health for women who are undergoing this process, health care professionals have to consider their feelings, helping them cope with the guilt and relieve negative emotions [12]. Likewise, the fact that caring for bereaved parents brings additional stress to the health care staff must be taken into consideration [5]. It is difficult for the staff to deal with negative outcomes in maternity wards, especially when there are no specific institutional protocols [13]. To be able to offer a better assistance, the professional also needs comfort [5], training, debriefing and professional support [2].

Guidelines and regimentation regarding pregnancy loss, stillbirth or baby death aren´t a new issue. In the UK, Sands (Stillbirth & Neonatal Death Charity)—a charity^1^ that works with grieving families and health care professionals published a very complete document [14] about this issue. Canada [15] and Australia & New Zealand [16], among other countries, also have their own and specific guidelines and France^2^ has specific laws. Those documents recognize the importance of late children, and ensures this care has permanent effects [1].

Until now, Brazil had sparse literature about this subject. Recently, a book based on the Canadian guideline which orients health care providers was published [17]. Research work was also conducted: a study about complicated grief comparing Brazilian and Canadian women who had lost their babies found that Canadians undergo a less complicated bereavement process than Brazilian women, suggesting that professional supporting bereavement groups, which is an incipient culture in Brazil, could had made de difference [18].

A recent Brazilian study brought to light the unrecognizable pain of fathers that experienced perinatal losses [19], discussing the male perspective. Another study from the same group highlighted lactation in the context of perinatal loss [20]. The latter is an important issue in Brazil, once pharmacological lactation suppression is usually the only available option offered to women. Preliminary discussion of The Perinatal Bereavement Project led to a publication in a nationwide circulation newspaper including several narratives of obstacles grieving mothers came across while trying to donate breastmilk^3^. Both the media report and the narratives from the book “Como Lidar: Luto Perinatal” [17] surfaced women´s understanding that donating breastmilk could help them cope with the loss of their babies.

In the 2000 document “Managing Complications in Pregnancy and Childbirth: A guide for midwives and doctors” (reprinted in 2007), the WHO presented principles to be considered when a baby dies, such as to avoid maternal sedation, to encourage women seeing and holding their babies, to collect babies’ mementos, among others [21].

In 2019, the action “Why we need to talk about losing a baby” was launched [22], aiming to turn miscarriage, stillbirth and neonatal death visible worldwide, as well as to depict the need for best practices and skilled professional healthcare. The initiative was grounded on women´s experiences of perinatal losses and epidemiological data, and proposed the end of “the unacceptable stigma and shame women face after baby loss” [22], claiming for empathy, respect and support during the care for those women. This is the beginning of a very important conversation about supportive guidelines for families experiencing stillbirth and neonatal death.

The development of evidence-based protocols may positively impact the health of parents who underwent perinatal loss. This protocol must be grounded on evidence provided by the hospital and health care professionals, and it must focus on creating memories regarding the late baby. A North American study examined 40 women who had a miscarriage between 12 and 20 weeks, and received support based on mediating protocols [23]. After the interventions, they felt diminished despair and reported feeling cared for and assisted. The findings may probably apply to pregnancy losses regardless of gestational age.

When a baby dies or a pregnancy is lost, parents experience multiple losses, such as the plans they have made for the live baby, and the dreams and expectations they had towards growing a family. Death also alters parents’ previous expectations about parenthood [15]. If we look to parents’ bereavement through the lens of Rando’s theory (1993) [24], “The Six ‘R’ Processes of Mourning”, we can find in the third process, “recollecting and re-experiencing the deceased and the relationship” huge difficulties for parents, due to the scarce or even absent actual memories of the baby who died, or of the lost pregnancy [15]. With this in mind, collecting memories is a key point within comprehensive perinatal bereavement guidelines.

Additionally, performing farewell rituals and facing grief in a realistic way are key aspects to cope with death: “Seeing and holding a live baby right after the birth is a normal parental response. Seeing and holding a stillborn baby is also a normal response, and there is much evidence showing that it can be a valuable and cherished experience” [25]. A 2015 systematic review analyzed health outcomes associated with parents who could see and hold their stillborn children. Results revealed that allowing families to have physical contact with their children is beneficial, which opposes previous concepts by which health care professionals should discourage such behavior [26].

Locally, there is evidence in national studies corroborating the importance of having contact with the baby, even after death. Parents mentioned the wish to hold the child close. They also reported that their pain was diminished due to this incomparable and fundamental experience [27]. Another national investigation concludes that rituals that included naming, seeing and touching the baby, as well as having funeral services, contributed to a healthy mourning [28].

In order for health care professionals to guarantee adequate assistance to bereaved parents, it is necessary that they are emotionally and technically trained and equipped [18]. Dealing with women suffering from the loss of a child can pose an enormous challenge, considering the cultural and personal uniqueness of each one of them [29–32]. Notably, taking care of the team enables the preservation of the continuity and quality of future care in situations of loss [31, 32].

### Research question

Do bereavement guidelines designed to assist parents who are experiencing stillbirth or neonatal death in a Brazilian childbirth facility promote supportive care that provides a healthy bereavement experience for the woman and her family?

### Underlying hypotheses

This investigation presupposes that local institutional protocols developed from bereavement-supportive guidelines may offer better assistance in the perinatal loss scenarios. It is based on the assumption that the physical and emotional care, as well as the moments spent with the baby and the collection of mementos, enhance the chances of a healthy bereavement experience.

### Conceptual framework

**Fig. 1:**
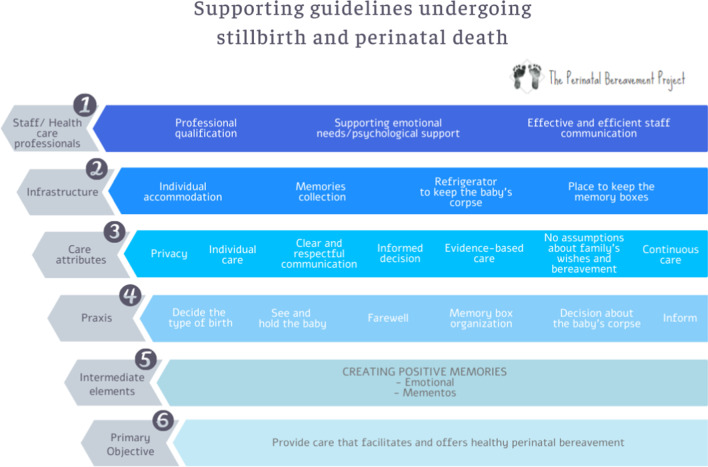
Supporting guidelines: stillbirth and perinatal death

The document “Pregnancy loss and the death of a baby: Guidelines for professionals—4th Edition” [14] published by SANDS was used to outline the conceptual framework for the present study. The authors considered that the main prerequisites for offering supportive care to bereaved parents are time, training and support. Good communication, shared decision and individual care are important elements to provide high quality grieving assistance. These are the grounding aspects for supporting guidelines and, for this reason, are on the top of the conceptual theoretical matrix, according to Fig. 1.

Initially, the first level of the matrix contemplates the work with staff and health care professionals. It involves three elements concerning professional qualification, meeting professionals’ emotional needs (including psychological support) and an effective and efficient staff communication. Considering the Brazilian scenario, where guidelines and orientation are needed, the following tripod is significantly important to outline a starting point for planning a supportive bereavement guideline: (1) prioritize initial capacitation regarding the guidelines and situation management; offer continuous development to both qualified and novice staff members so all are aligned with the care model to be followed; (2) understand that the health care professionals are above all humans and, therefore, subjected to grief, death and bereavement in their professional and private lives. They may also be more affected by a grief situation while assisting patients. Consequently, they need institutional space to address their own emotional needs. With this in mind, the Bereavement Professional is allocated to provide care and sensitive listening without any judgment. This action aims to meet the professional/staff needs; (3) offer good communication, among professionals and among staffs who will also provide the families good communication. This is the core aspect to ensure parents are being given what they need during the bereavement process. Good communication consists of establishing and following protocols with which the whole staff is aligned.

The second level of the matrix is related to the infrastructure of the health care establishment that will provide the family physical and emotional memories of the baby. Some of the infrastructure items consist of the family's individual accommodation, resources to collect memories, a refrigerator to preserve the corpse and a local to keep the memory boxes. The aforementioned items are fundamental and most Brazilian maternity wards need to restructure according to the following: (1) individual accommodation is one of the issues Brazilian grieving parents mention the most. It protects them from curiosity; it prevents them from having to share the grieving moment with other families who are celebrating the arrival of a healthy baby next to them; it also gives them privacy to meet their child moments before sending them to funeral rituals. Even with logistics challenges in Brazilian childbirth facilities, some privacy must be considered and offered; (2) resources to collect mementos inside the maternity ward, such as a camera to take pictures of the baby and the family or material to register the handprints and footprints, and a lock of the baby’s hair, when available. A box to keep the items is important for the process of creating memories and should be offered to the family before hospital discharge; (3) refrigerator to preserve the corpse is one of the least useful items, but it might be essential at some point.

In childbirth facilities where complex cases are assisted, many women need sedation or are unconscious for a period of time (days or even weeks). When the woman is awake, after being unconscious during birth and the first hours or days after childbirth, the impact of the news may be devastating, especially when the baby has already been buried or cremated. In such situations, having the chance to keep the baby so the mother can recover from sedation/unconsciousness may have a positive outcome for her mental health; (4) having a place to keep the memory boxes that were not taken by the family after hospital discharge, is also important. Chances are these families will return the following weeks or months to take the boxes with them once decision may change over time.

Health care attributes are on the third level of the matrix. They highlight the essential aspects of the care given to parents: privacy (previously mentioned); individual care, that adapts assistance to a family’s physical, social and emotional needs. These adjustments are based on beforehand locally developed alignments of care. This is a delicate matter and health care professionals have to observe ethical and non-judgmental values when it comes to the family’s decisions, beliefs and feelings. It is also challenging since the professionals themselves are personally and individually affected. They have their own mechanisms to deal with grief and that has to be considered, as discussed in the previous level of the matrix. Our suggestion to solve this problem is teamwork. Therefore, professionals are supported by the health care establishment and the structured protocols and may follow the pre-established recommendations. Informed and conscious decisions, evidence-based care, pre-established institutional protocols and uniform technical behavior are part of this approach to produce safe assistance and continuous care.

The praxis is described on the fourth level. It highlights the need to decide the birth as the first move. This consists of an issue regarding vaginal birth versus cesarean section, since the Brazilian scenario has worrying numbers of C-sections. In some cases, when the woman’s life is at stake, cesarean sections are actually needed. However, in most stillbirth cases, vaginal births are the safest choice.

The mode of birth is a mother’s decision. Nevertheless, health care professionals should explain the risks and benefits of each option. Women should have the chance to experience labor and vaginal birth. This may help them start grieving, since time, hormonal and physical questions may be addressed. Moreover, when the woman opts for a vaginal birth, she will actively participate in the biological parturition process and she will be able to see and hold the baby, as well as have a farewell moment, since childbirth. Additionally, she will probably have a better physical condition to participate on rituals (funeral, burial ceremonies or cremation) and to get pregnant again sooner, if she wishes. Lastly, C-sections increase risks in future pregnancies and childbirth when compared with vaginal birth. Thus, all of the staff’s efforts should involve providing the best health care, avoiding physical and psychological risks for the mother. As a result, vaginal birth tends to be a better option whenever possible, but sometimes, especially when there is a psychological trauma, a C-section should be considered. In any case, if a C-section is performed, holding the baby is recommended and should be encouraged. In all cases, the staff will organize the baby’s memory box and help with the decision-making process regarding the baby’s corpse. It is also advisable, whenever possible, to have written plans for labor and postpartum and for all decisions related to the baby.

In Brazil, post-mortem examinations are hard to be run. Yet, whenever possible, they should be suggested by health care providers. Finding the cause of death may help the family mourn, even though clearly stating diagnosis is infrequent. Funerals, burial ceremonies or cremations should be postponed so necroscopic examinations may be run. Notably, even when the death occurs before fetal viability (20 weeks and/or 500 g), the family may opt for funerals, according to Brazilian law and the Federal Council of Medicine technical reports [33]; it is essential that the family have all the information they asked for (e.g., procedures, clinical questions), which will support their decision-making.

The intermediate elements of the matrix transcend the objective aspects described here. They include creating positive memories that are associated with the baby and its death, which is challenging and complex. For this, we recommend personalizing the approach and respecting the woman’s decisions, aiming to lessen anxiety and uncertainties. An institutional protocol that is uniform and well-structured is essential. An open dialogue among all the people involved in the care and emotional support given by the professionals are fundamental points.

The end of the matrix regards the primary objective of this proposal: to offer care that facilitates healthy perinatal bereavement. This may be achieved when all the measures mentioned above are transversally contemplated.

## Rationale for developing supporting guidelines for stillbirth and perinatal death in Brazil

So far, there are no supportive guidelines for stillbirth or neonatal death in Brazil. Health care services and health care professionals deal with each situation according to their own belief. Sometimes the professional offers care based on what suits him/her, since dealing with bereavement patients promotes stress and anguish. Additionally, there is no institutional support available to help hospital staff care for grieving patients. Health care assistance frequently ends fast and close contact is avoided, once dealing with grieving families is often challenging. Therefore, outlining supportive guidelines is urgent. Health care establishments will be able to create local protocols to care for families who are going to experience the death of a baby. Likewise, these protocols will lessen health care professionals’ stress.

Supporting guidelines are supposed to be used as a reference to those who want to adapt and implement them in different health care contexts and scenarios and use them locally. These tools may promote the structure of the care given to families who are experiencing perinatal loss. Notwithstanding, the focus is not only the pregnant or puerperal women’s well-being, but also the health care professional’s welfare.

## Objectives

The primary objective of The Perinatal Bereavement Project is to assess the effects of international bereavement guidelines adapted to the Brazilian context in the mental health and grief experience of mothers who have undergone a perinatal loss. The secondary objective is to verify the prevalence of postpartum depression, anxiety, stress and grief adaptation symptoms, as well as assess the care received in two different moments: before and after the health care professionals are trained and maternity wards are prepared.

## Methods

### Study design

This is a quasi-experimental study that uses qualitative and quantitative methods. It also uses a before-and-after analytical approach.

### Setting

#### Data collection procedures

##### Study phases:

Preparation: This is the phase where the meetings with the maternity ward staff will take place and the Reference Professionals for each maternity will be assigned. Reference Professionals will be responsible for keeping in touch with the researchers who will answer any questions about the project; for organizing training/courses; informing the list of stillbirth and neonatal death cases; etc. They are also going to recommend a Bereavement Professional for the maternity ward. The Bereavement Professional is responsible for bereavement issues regarding the families and the staff. The same person may be both the Reference Professional and the Bereavement Professional. Reference Professionals may be any professional who works at the maternity ward, and has significant experience with the maternity ward routine and procedures, and with the staff. There could be two, both responsible for managing, professional training and guiding. They will be in contact with the family when the allocated professional is absent or ask for guidance. They should be a physician, a nurse or a midwife.

The Bereavement Professional should have experience and skills to offer emotional and technical support to the staff. They may be available to support the families as well. Accordingly, the maternity ward managers may decide if one or two Bereavement Professionals will suffice. Ideally, a psychologist should be the Bereavement Professional, but professionals with further skills may assume the position. The Bereavement Professional will be trained in theoretical and practical contents to offer assistance as required.

The time has come to promote awareness for managers, Bereavement and Reference Professionals regarding perinatal mourning processes. It is also time to elaborate what in Brazil is called Standardized Operational Procedure (SOP) (in Portuguese, “*Procedimento Operacional Padrão*”—POP), and professional qualification.

### Pre-intervention

This phase will consist of two actions: (a) meetings with the focus group and (b) interviews with the women.

- *The pre-intervention focus group*
  During the pre-intervention focus group, an informed consent form will be signed, and participants will answer sociodemographic and work experience questionnaires. The meeting will last no longer than 1h30min.
- *The pre-intervention interview with women*

The pre-intervention interview with the women will be carried out by one of the researchers, based on the stillbirth and neonatal death list provided by the Reference Professional from the maternities ward. They will get in touch to schedule an in-person interview in a place to be defined. Information to verify inclusion and exclusion criteria will be covered. After inclusion criteria are met, the women will receive information concerning the study and will be invited to participate. If they accept, the in-person interview will be scheduled. During this meeting, the informed consent form will be signed. Afterwards, mental health scales will be applied. A qualitative interview will cover socioeconomic, demographic and behavioral data regarding the loss the woman went through.

### Intervention

**Fig. 2:**
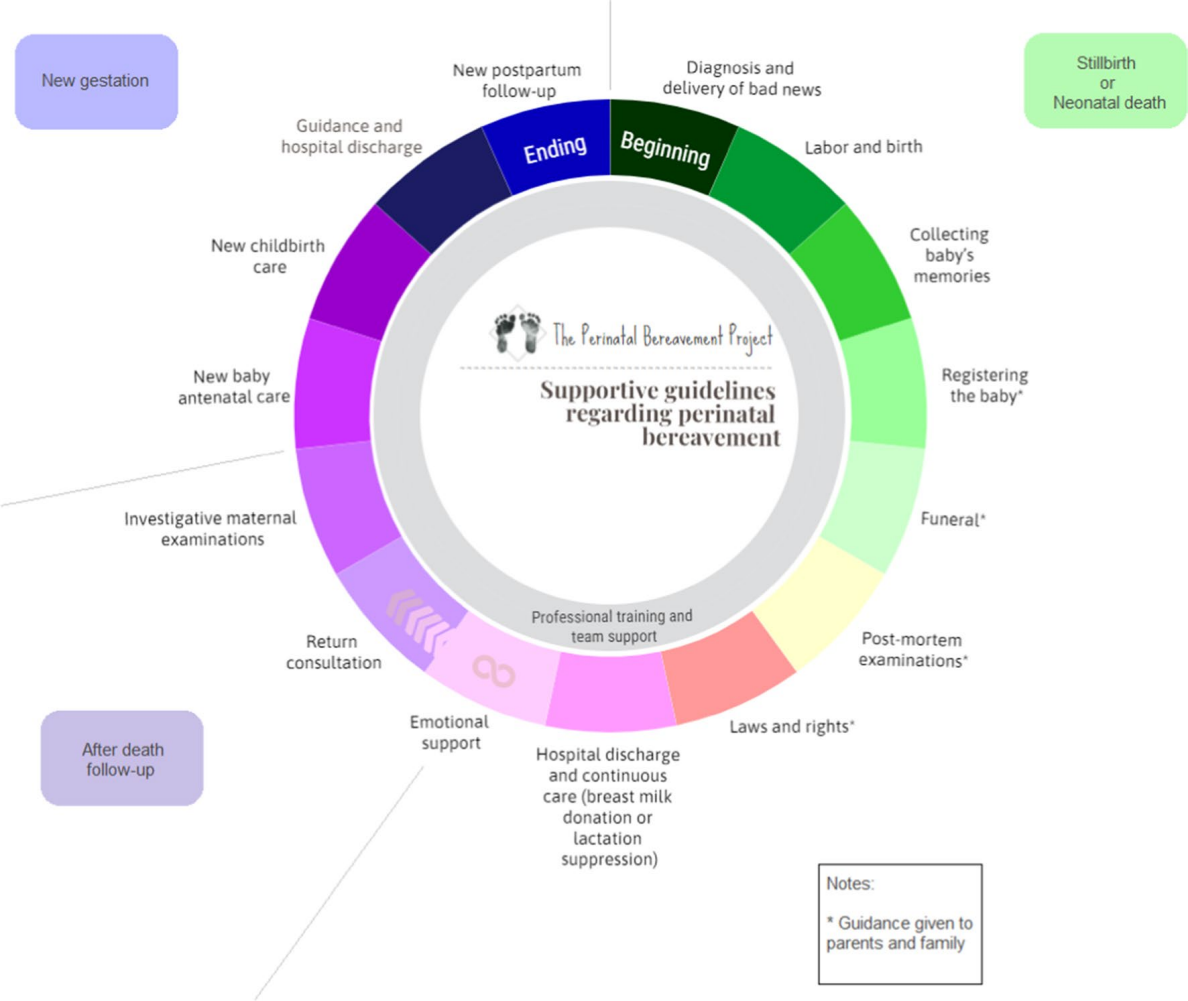
A proposal for supportive guidelines regarding stillbirth and neonatal death

The intervention will be based on two publications: the Brazilian book “*Como lidar—luto perinatal: acolhimento em situações de perda gestacional e neonatal”* [17], based on the Canadian “Guidelines for health care professionals experiencing a perinatal loss” [15] and the manual provided by SANDS (Stillbirth & Neonatal Death Charity) “Pregnancy loss and the death of a baby: Guidelines for professionals” [14]. Both offer a framework to coordinate important actions to assist families as shown in Fig. 2.

Additional material will be published during the investigation and produced along the process. Based on the British manual aforementioned, the initial content will be broadened. This material will be available on www.saude.fmrp.usp.br/lutoperinatal. All documents staff should use during hospitalization (Perinatal loss and Memory creation files) will also be available on the same site. The perinatal loss file will hold relevant information about women’s health and the guideline step-by-step. It will help professionals share information. The memory creating file will provide guidance about the steps taken regarding the baby memory box. A summary of the main actions regarding the supportive guidelines is available on Table 1.

**Table 1:** Summary of the main actions on supportive guidelines for families experiencing stillbirth and neonatal death

Summary of supportive guidelines regarding perinatal bereavement	
How to prepare the family to see the baby:	Clean the baby (do not bathe him/her); Dress the baby with diapers, socks, hat, clothes, blanket etc.; Follow the same maternity ward protocol to bring alive babies to see their mothers; Call the baby by his/her name; Follow them up but keep some distance. Never let them unassisted
What should be provided for the memory box:	Hair lock; Handprint and footprint; Placenta stamp (optional); Baby’s pictures; Baby’s first clothes; Baby’s bracelet/identification tag; Cards and letters written by the staff; Leaflets about the bereavement process and bereavement support groups
Which written guidance should be provided:	All information about funeral services and local civil registry office; Information about how to deal with breast milk production; Suppressing lactation Breast milk donation Post-discharge care; Medical appointments and additional exams for further investigation (if necessary); Bereavement support group; Psychological services
What to do to provide continuous care:	Discuss family planning; Schedule regular appointments to assess mental and physical health, and discuss new findings about what happened to the baby (when applicable); In the case of a new gestation, follow health care guidelines for pregnant women who had previously experienced the loss of a baby; Arrange to assist woman during the new puerperium
Guidelines for assisting pregnant women who had previously experienced the loss of a baby:	Remember: this is no longer an ordinary pregnancy, and may be considered a risk pregnancy from the mental health point of view; The previous loss may impact the following bereaved mother’s gestation, from antenatal care through puerperium and breastfeeding; Fear and complaints are expected responses and must be acknowledged; Some parents may prefer to be cared for in a different hospital or by different staff members, which should not be a problem; Mental health screening should be available for both parents; Specialized mental health support or psychological appointments should be encouraged at any time during pregnancy and postpartum; Consider avoiding standard antenatal classes, prioritizing individual preparation sessions for labor, birth and caring for a newborn baby; A special sticker can be used on both notice board and at room’s door wherein a bereaved mother is in labor, so that the staff is aware of her condition; Encourage the writing of a birth plan, so that parents’ preferences can be easily shared with all staff during labor, birth and postpartum Consider scheduling extra and longer antenatal appointments. Extra screening options should be considered whenever necessary or demanded; Set aside a weekly day or time at your institution for pregnant women who had previous perinatal losses to seek assistance if they have questions about their baby's well-being or their pregnancies´ normality; A C-section without clinical recommendation may be considered, when fear from natural birth could not be dealt with after several psychological and communication interventions, as long as the women acknowledge associated risks
What to do when there is: *Stillbirth* *Neonatal death or imminent neonatal death* *Severe malformation (incompatible with life or high death probability)*	1. Assure the mother/family privacy; 2. Deliver bad news properly; 3. Give the family all requested and necessary information; 4. Encourage the preparation of a Care Plan for the women and the baby. This Care Plan includes a birth plan, whenever possible, and a palliative care for the baby, whenever necessary; 5. Choose and prepare a private room, to avoid contact with other mothers and their babies; 6. Apply special stickers on both notice board and at the bereaved mothers’ room door, so that the staff is aware of her condition; 7. Avoid sedation; 8. Respect initial plans – whenever possible; 9. Encourage the mother/family to see the baby; 10. Encourage the mother/family to touch the baby, and to spend time with her/him; 11. Provide the memory box

Regarding the model Training of Trainers, an expert teaches a professional who works in a health care establishment. The one who receives the instructions will teach the others. Therefore, after the implementation of the Project, the qualified professional will continue providing refreshment courses to those who need it. For this specific project, the expert will be the researcher. Another professional will participate in the qualification process and will be in charge of the regional staff after the expert finalizes the project.

The training method consists of the following events:

• Perinatal Bereavement Event—training: An in-person event that will be organized with the Regional Health Department XIII. Although it will be focused on specific professionals, managers and Reference Professionals, it will be open to all local professionals. Estimated duration: 3 h.
• Reference Professional Meeting: This in-person event aims to prepare and help the Reference Professionals get ready to start the project and write the Standard Operational Procedure (SOP). They will also plan the qualification of the professionals in their health care establishments. Participants: professionals indicated by maternity ward managers (physicians, nurses and psychologists). Estimated duration: 3 h.
• Bereavement Professional Meeting: An in-person event aiming to offer the Bereavement Professionals theoretical content about bereavement, the family’s and professional’s points of view, so they may carry out their functions. Participants: professionals indicated by maternity ward managers (physicians, nurses, psychologists and social work professionals). Estimated duration: 4 h.
• Coordination meeting: An in-person meeting will be held in each maternity before the staffs are formed. It aims to raise professionals’ and team work awareness on the subject. A video will be produced and will be available to the professionals who could not be present.
• Support network to answer any questions and guide professionals involved: During the whole time the project is being carried out, the researchers and the health regional representative can be reached by phone, WhatsApp (general group chat, maternity ward group chats and private messages);
• In-person and virtual follow-up will be provided until the end of the project.

Among the actions in accordance with the training methodology, some items will be given to the families. Additionally, the following are to be given to the staff: A box to keep the memories, a polaroid camera and polaroid paper, card for handprints and footprints, a small plastic bag to keep the hair lock, a big plastic bag to keep baby’s clothing items, a blanket that is used when the baby is too small for conventional clothes.

### Post-intervention

This phase consists of two actions: interviews with each woman in the post-intervention group and meetings with the post-intervention focus groups.

One focus group consists of the nurses in each maternity ward and the other is formed by the managers. The interviews and the meeting will mirror the steps taken in the pre-intervention phase.

## Study participants

### Participants

Women who experienced stillbirth and neonatal death in one of the four public maternity wards in Ribeirão Preto City, in São Paulo State, southeast region of Brazil. Health care professionals, employees and managers of the respective maternity wards will also participate.

#### a) Participants and eligibility criteria

All four public maternity wards in Ribeirão Preto were invited to participate in this project and all of them accepted.

No exclusion criteria were outlined for health care professionals, employees and managers.

The *inclusion criteria* for women are:

- To live in one of the municipalities of the Ribeirão Preto region (XIII Regional Health Department, São Paulo State)
- Have experienced a stillbirth or a perinatal death during the estimated period^1^;
- Have faced the death of her baby in the following circumstances:
- Pregnancy that lasted more than 20 complete weeks;
- Had a baby weighing at least 500 g;
- Had a baby that died no later than twenty-eight days after the birth (neonatal death).

Women *exclusion criteria* are to not understand Portuguese and have severe mental health impairment.

The main difference between the pre- and post-intervention groups is the training process health care professionals will receive, together with preparing the maternity ward to implement local protocol. It is estimated that women who had a baby born in the pre-intervention group did not receive a care based on the supportive guidelines regarding perinatal bereavement.

The first 20 women from each group who accept to take part in this study will be interviewed.

#### B) Research instruments and data sources

Data will be collected from women interviewed and from the focus group offered to professionals. We will use a questionnaire specially designed for this project, as well as validated tools used in previous mental health and bereavement studies. Pilot interviews were performed as a way to validate the questionnaire and organize the interview process.

Three scales, that were adapted and validated to Brazilian Portuguese, will be applied:

- *Perinatal Grief Scale (PGS)*
  The PGS presents 33 self-applicable psychometric assertions, divided into three subscales, related to perinatal grief adaptation symptomatology. Involves a five-point Likert Scale ranging from “strongly disagree” to “strongly agree”. The cut-off score that we will use is 90, as validated by Paris et al. [34, 35] for the Brazilian population.
- *Edinburgh Postpartum Depression Scale (EPDS)*
  The EPDS is a set of 10 self-applicable screening questions, each of them with four multiple choice responses, which indicates the intensity of depressive signs and symptoms that are present during the last seven days before the application. The cut-off score that suggests the woman is probably depressed (or with depression symptoms) is ≥ 11, as validated by Santos for the Brazilian population [35].
- *Depression Anxiety Stress Scale-21* (DASS-21)
- DASS-21 is a self-applicable scale with seven items each. It assesses depression, stress and anxiety, considering the previous week. The answers are given in a four-point Likert Scale that vary from 0 (totally disagree) to 3 (totally agree). Score variations correspond to symptom levels such as “normal” and “severe”. The global scores for the three constructs (depression, stress and anxiety) will be calculated as validated by Vignola et al. for the Brazilian population [36].

A sociodemographic questionnaire designed to collect personal data, behavioral information, medical history and information about care received will also be applied.

Each maternity ward will assign the *Reference Professionals* and the *Bereavement Professionals* among their employees.

Data will be provided by each health care establishment. They will send a list containing the stillbirth and neonatal death list, provided by the respective Reference Professionals. The researcher will contact each candidate to verify eligibility for this study, inviting them to participate and scheduling a meeting.

Focus groups will be conducted by the researchers, and the health care professionals who will participate will be contacted by the Reference Professional of each maternity ward.

## Primary and secondary outcomes

### Primary outcomes

A composite of a grief state (grief adaptation symptoms) and rates of depression, stress and anxiety.

### Secondary outcomes

- Absence from work due to bereavement
- Psychiatric treatment due to bereavement
- Maternal admission to psychiatric health facilities
- Suicide attempt
- Suicide

### Variables

#### Variables of interest (***Table ******2***)

**Table 2:** Variables of interest

	Variables of interest	Data source	Statistical methods
Sociodemographic data:	Age; Marital Status; Schooling; Socioeconomic status; Skin color; Job; Religion	Questionnaire	Descriptive analysis Frequency analysis
Characteristics/ Personal and behavioral information	Recreational activities (sports and artistic activities); Practices sports; Uses drugs (alcohol, cigarettes and/or illegal drugs; Is under medication and/or psychiatric treatment; Participates in religious groups; Undergoes psychotherapy; Participates in bereavement support groups	Questionnaire	Descriptive analysis Frequency analysis
Health history	Gestational age; Miscarriage, abortion, stillbirth or neonatal previous deaths; Illnesses before gestation; Illnesses in the present gestation; Fetal malformation; Childbirth complications	Questionnaire	Descriptive analysis Frequency analysis
Intervention assessment data	Satisfaction with the assistance given	Questionnaire	Descriptive analysis Frequency analysis
	Perception level regarding anxiety, stress, depression and port-partum depression	Edinburgh Postpartum Depression Scale Depression Anxiety Stress Scale-21	Descriptive analysis Frequency analysis
	Perception level regarding signals (symptoms and feelings) of bereavement	Perinatal Grief Scale	Descriptive analysis Frequency analysis
	Difficulties with life routine resumption	Perinatal Grief Scale	Descriptive analysis Frequency analysis

#### Bias (*Table****3***)

**Table 3:** Possible Issues and Solutions

	Possible Biases	Possible Solutions
1	Health care professionals may disregard important orientations or may have difficulties in changing their routine in the beginning	The Reference Professional will guide them from the beginning of the project and so on
2	A mistake regarding gestational age or baby weight on the maternity ward reports may include/exclude a woman from the sample	The information regarding the baby (birth, death) will be checked with the mother in the first contact
3	Mistakes made while filling out the documents created by this project	The Reference Professional will help filling out the documents and will follow up the case discussions
4	The mother/family does not authorize collection of some items listed in the project (picture, for instance)	The Reference Professional and the Bereavement Professional will manage this situation and consider alternatives to solve this matter. If necessary, researchers will assist them
5	During interview, a woman from the pre-intervention group reports that she has received care based on the supportive guidelines regarding perinatal bereavement	During the first contact, she will be asked for some information that may help avoid this bias

### Calculation of sample size or available sample sufficiency

Considering the financial and human resources for the project as well as the low prevalence of events of interest, we chose to establish the sample size by convenience (pragmatic sample size). In this context, time to select women who will participate was the decisive factor for sample size. Sample size in one study in the same field was 44. Given the amount of available resources, we fixed the post-intervention time in six months. Thus, given the selection period of time, 40 women is the number estimated (20 before intervention and 20 after intervention).

### Ethical and equity aspects

This study is in accordance with the Helsinki Declaration [37] and follows the guidelines and norms of the National Health Council [38] order number 4666/12. The data collected (interviews, scales that were translated to Brazilian Portuguese, scales that were validated by Brazilian investigations) will be exclusively used for academic research. The identity and privacy of the participants will be respected.

Each participant (women who lost their child, professional and managers) will receive two copies of the informed consent form. They will provide information about the study and the research staff’s phone number and emails. This document will be read and signed in case of agreement. The participant and the researcher will keep one copy.

Women who present any risk to their mental health will be forwarded to psychological/psychiatric assistance according to their city of origin. In case they are diagnosed by the health care establishment staff, the respective protocols will be followed. Should diagnosis be known during the second interview, the researcher will send the participant’s personal information (name, birthday, national health identification, mother’s name, and hometown) to the XIII Regional Health Department, under the care of the respective health care professional. This professional will take the appropriate measures so the case may be followed up accordingly.

Notably, this study will not offer participants any significant risks. The results will be applied to benefit perinatal loss assistance all over the country. Additionally, this investigation aims to promote equity, which might be reached when local protocols are used. They will take into consideration the most important aspects of the supportive guidelines, as the assistance provided is personalized, respectful and dignifying. Thus, the patient’s physical and emotional necessities are addressed.

#### Project management

The project management will be carried out by University of São Paulo researchers, as well as the Steering Committee and the Security and Data Monitoring Committees. The Technical Advisory committee will be held by a researcher from São Carlos Federal University and a specialist of “4 Estações Psychology Institute”^4^ a grief institute in the city of São Paulo. The data analysis committee will be held by all researchers.

### Data management and analysis

#### Data analysis plan

The data will be analyzed by the project staff and, whenever necessary, by experts. The analysis plan as well as a detailed plan for statistical analyses will be designed before data collection starts. Qualitative thematic analysis will be applied on the data from the questionnaire, considering the pre-determined categories and new categories the analysis will provide. The data regarding the focus groups will be treated likewise.

#### Descriptive analysis

**Fig. 3:**
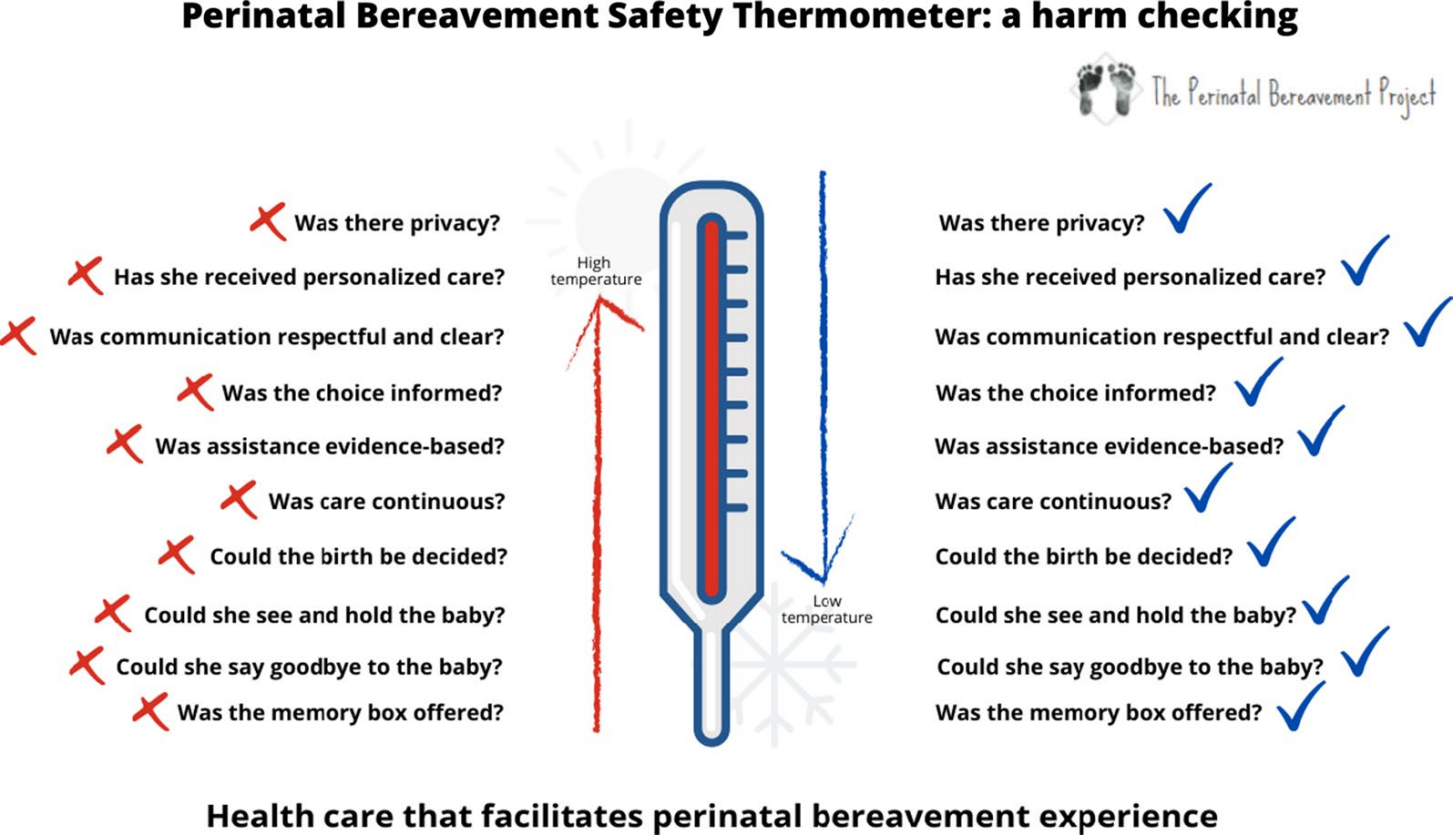
Perinatal Bereavement Safety Thermometer: Harm Checking

Descriptive analysis will be based on the qualitative thematic analysis and harm frequency identified by *The Perinatal Bereavement Safety Thermometer* developed for this investigation (Fig. 3).

*The perinatal bereavement safety thermometer* provides a harm checking of 10 important aspects that impacts the maternal mental health and the grieving process. It is based on the British *Safety Thermometer*s tools developed by the National Health Service (NHS) created for harm checking in five different areas [39].

According to the tool proposed for this study, low temperatures of harm offer a better chance for a healthy perinatal bereavement, once it promotes better memories for the mourning process and the bereavement experience. In the same way, we assumed that an ideal health care assistance should avoid situations that may be interpreted as harm. All the ten items predicted on this tool are considered central and take part on British [14] and/or Canadian [15] guidelines.

Descriptive analysis will be performed to reveal harm frequencies (Fig. 3) caused by health care assistance that did not observe the supporting guidelines for perinatal loss. This analysis will also show the relation between these harms and the findings provided by the three tools used to verify women’s mental health and grieving process.

Thematic analysis will also be carried out by exploring the findings related to the objectives of this investigation. They will be compared with each group and among the groups. The analysis will consider cultural and gender issues. It will also contemplate institutional specificities that will guide the implementation of the protocol in each maternity ward involved in the project.

The qualitative thematic analysis was chosen due to the fact that it is possible to map, analyze and identify pattern (topics) in the data. It has the following steps: 1) data organization; 2) pattern, topic and category mapping; 3) emerging hypotheses testing and 4) alternative explanations gathered from (pre-intervention, post-intervention and focus) groups, as well as from comparative analysis of the groups.

#### Quantitative analysis

A detailed plan for statistical analysis will be developed by the researchers before data collection starts. A modelling plan will be developed and implemented by a team of experts that includes biostatisticians, psychologists and obstetricians.

Simple frequencies will be calculated, and a comparison method will be used (T Student or Chi Square tests).

#### Publishing the findings and results dissemination plan

Our findings will be published in peer-reviewed scientific journals (in English and Brazilian Portuguese). Moreover, the results will be locally shared with those involved in the design of the project, as a way to promote debates and reflections on the present praxis. It will also help create new measures for better outcomes in the future, which will affect the families who will experience stillbirth and neonatal death.

## Discussion

### Projection of the main findings

The approach to women and families who lost their babies during gestation, labor or post-partum period must follow a specific supportive guideline, which has to be locally adapted by Brazilian maternity wards. The way this issue has been addressed by health care professionals and the maternity wards may impact the process either positively or negatively. When negative emotions prevail, the technical approach might produce long-lasting, adverse psychological effects that might remain for a long time, sometimes even a lifetime.

The development of Brazilian supportive guidelines for perinatal loss, based on international experiences, may fill in an important gap regarding women’s health. It will be contemplated from a perspective focused on women’s sustainable development of wellbeing and empowerment. Additionally, our guidelines cover professional qualification. The staff that will be involved with this specific kind of care will interact to elaborate situational and contextualized scenarios to promote a better relationship between health care professional and patient.

The qualification of the bereavement professional is another relevant aspect of these guidelines. The Bereavement Professional will not only help professionals deal with specific and complex questions regarding grief, but also assist grieving families. He/she will address the private and individual matters of staff and professionals. These questions may be raised along with the assistance given to the families during the bereavement process. Among the Bereavement Professional’s tasks are the reorganization of staff, psychological support and empathic listening, whenever necessary.

### The contribution of this study to assist stillbirth and neonatal death

These supportive guidelines aim to assist women and families who are experiencing stillbirth and neonatal death. This assistance is grounded on empathy and efficiency during this challenging time. Moreover, it proposes continuity of care, including comforting the woman and her family, assisting the birth and designing care procedures after hospital discharge. This might contribute to the woman’s wellbeing and, consequently, her quality of life, as well as her mental and physical health. Likewise, the creation of standardized hospital procedures may provide the professionals more satisfaction at work, since occupational stress and communication anxiety are reduced, and unwanted obstetric outcomes are better managed. Notably, these guidelines will offer practical orientation towards technical routines related to the death of a baby during gestation. It will include corpse handling, memory box, among other routines.

### Obstetric applicability

The applicability of these guidelines will result in a pilot study. This way, the guidelines might be applied in similar scenarios. Moreover, based on our guidelines, other maternity wards or organizations will be able to design their own proposals for perinatal loss approach. They may use our findings to contemplate regional differences regarding perinatal bereavement, such as the ones in indigenous communities, *quilombolas*, favelas, and other groups.

### Anticipating main problems and proposing solutions

Dealing with death in childbirth facilities is a delicate situation. It might cause discomfort and privacy disruption in patients and professionals. We will try to minimize these problems and offer a clear outline of the objectives of the present investigation. We will highlight the benefits the project will bring to the family who is experiencing this trauma, and to the ones who will be in this situation in the future.

All participants will be granted the right to privacy throughout the research project. They may also stop participating any time they want. Authors/researchers who will deal directly with the participants are prepared to listen to them empathically and professionally and avoid any kind of embarrassment and discomfort this investigation may cause.

Finally, any problem or challenge professionals might have regarding bereavement will be addressed kindly, inside the health care establishment and, whenever necessary, will be handled by a qualified psychologist.

Some of the limitations and difficulties in this study can be accessed on Table 4.

**Table 4:** Forecasting Difficulties: Mitigation Strategies

	Difficulties	Strategies to mitigate them
1	Participants may display strong emotions, such as sorrow, despair, anger, etc	Interviewers will be prepared for these situations and will interrupt and reschedule the interview if necessary
2	Participants may feel emotionally uncomfortable to share their experiences and feelings regarding the loss of the baby, since they are mourning	The questionnaire is very detailed and the fundamental information to understand the assistance is based on yes/no questions
3	Health care professionals may be experiencing grief in their lives. Also, bereavement situations may trigger previous feelings related to losses that happened in their professional or private lives. This might compromise or limit the assistance	The Bereavement Professional will pay close attention to this problem and may support the professional and/or reorganize the staff and substitute this member
4	The maternity ward might not have the resources to follow the new guidelines for perinatal bereavement	Limited resources are addressed by this project. It is possible to adjust them accordingly
5	Professionals and employees may feel some discomfort or find the guidelines morbid	The Reference Professional will help the professionals and the staff adjust to the new routine. More adapted professionals may join the staff to help transition
6	Difficulties regarding continuous care after hospital discharge	The professional from Regional Health Department XIII (Woman Health) will follow up on the implementation of the project and will address this problem
7	The maternity ward does not want to share data (women’s telephone numbers, emails, etc.)	The Project management staff understand that in the event the maternity ward does not want to share data, it may be included in the staff training, but not in data collection. Training and data collection are not imbricated

### Quality control

The researchers will follow up the implementation of local protocols. This will be feasible during in-person meetings with the Reference Professionals and interactions to promote on-the-spot problem solving. The qualification of professionals and staff regarding the protocols will be monitored by the researchers all the way through the project.

### Data privacy

Data confidentiality will be protected during the project and afterwards, by the researchers and IT professionals involved in the research group. Access to the digitalized content is granted via encrypted password and data safety systems. Printed material will be avoided and/or destroyed after the project.

## Research sustainability

### Environmental impact estimates

The environmental impact of the present research will be calculated by the tool available at https://www.tjpr.jus.br/web/gestao-ambiental/calculadoraco2. The Environmental compensation plan will be designed with a local institution based in Ribeirão Preto that donates seedlings and resources to plant trees. In the future, based on the carbon dioxide emission, the seedlings will be planted according to the instructions given by the Ribeirão Preto City Hall. The number of trees will compensate the CO2 emissions that happened during this investigation.

The tree planting will happen during an event similar to ones that have already been held in other Brazilians cities—Araraquara (São Paulo State), Goiânia (Goiás State), Recife (Pernambuco State)—where the parents who had experienced stillbirth or neonatal deaths will plant a tree to honor the child they lost. Thus, a park/garden/groove of memories will be created. This event is intended to take place annually, so more parents can join those who have already planted their trees. It has a positive outcome considering not only the bereavement perspective, but also the environmental one.

## Footnotes

1. SANDS (Stillbirth & Neonatal Death Charity): https://www.sands.org.uk/
2. Circulaire interministérielle DGCL/DACS/DHOS/DGS/DGS/2009/182 du 19 juin 2009: https://web.archive.org/web/20130226185927/https://www.sante.gouv.fr/IMG/pdf/circulaire_182_190609.pdf
3. Women who lost babies in late pregnancy want to donate breast milk: https://www1.folha.uol.com.br/cotidiano/2019/10/mulheres-que-perderam-bebes-em-fase-final-da-gestacao-querem-opcao-de-doar-leite-materno.shtml
4. 4 Estações Psychology Institute / Instituto 4 Estações: https://www.4estacoes.com/

## Supplementary information

**Additional file 1**. Full Portuguese translation of *The perinatal bereavement project: development and evaluation of supportive guidelines for families experiencing stillbirth and neonatal death in Southeast Brazil—a quasi-experimental before-and-after study*.

## Data Availability

The datasets used and/or analyzed during the current study are included in this published article and supplementary information can be accessed upon reasonable request. The full Portuguese translation of this article is available as Additional file 1.

## Abbreviations

DASS-21: Depression Anxiety Stress Scale-21
PGS: Perinatal Grief Scale de Luto Perinatal
EPDS: Edinburgh Postpartum Depression Scale
SOP: Standard Operational Procedure
WHO: World Health Organization

## Footnotes

1 Due to the Covid-19 pandemic, the period for recruiting women will change.
