# Correction to: The perinatal bereavement project: development and evaluation of supportive guidelines for families experiencing stillbirth and neonatal death in Southeast Brazil—a quasi-experimental before-and-after study

**DOI:** 10.1186/s12978-021-01191-y

**Published:** 2021-07-05

**Authors:** Heloisa de Oliveira Salgado, Carla Betina Andreucci, Ana Clara Rezende Gomes, João Paulo Souza

**Affiliations:** 1 Department of Social Medicine, Ribeirão Preto Medical School, University of São Paulo, Avenida dos Bandeirantes, 3900, Monte Alegre, Ribeirão Preto, SP 14049-900 Brazil; 2 Department of Medicine - Center for Biological and Health Sciences, Federal University of São Carlos, Rod. Washington Luiz, s/n, São Carlos, 13565-905 Brazil

## Correction to: Reprod Health (2021) 18:5 https://doi.org/10.1186/s12978-020-01040-4

During the publication process of the original article [1] the Portuguese abstract, keywords and full Portuguese version were accidentally omitted during the publication process.

The Portuguese abstract, keywords and full Portuguese paper (Additional file 1) are available in this correction article. The original article has been updated. We apologize to the inconvenience caused to the authors and readers.

**RESUMO**

**Contexto** Uma assistência avaliada positivamente por mães e pais que passaram pela perda perinatal permite a criação de memórias físicas e afetivas do bebê e possuem efeitos positivos no processo de luto da família. Este estudo avaliará os efeitos de uma diretriz de acolhimento na saúde mental de mulheres em processo de luto perinatal e neonatal em maternidades públicas do município de Ribeirão Preto (SP, Brasil).** Método** Estudo de métodos mistos (quantitativo e qualitativo), quase-experimental (antes e depois). A intervenção é a implementação de diretrizes de acolhimento ao luto de mulheres que tiveram um natimorto ou óbito neonatal. Um total de quarenta mulheres serão incluídas. Vinte participantes serão avaliadas antes, e vinte após a implementação da diretriz de acolhimento nas instituições. Serão aplicadas três escalas e uma entrevista semiestruturada para avaliar os efeitos da diretriz. Profissionais da saúde e gestores serão convidados a participar de grupos focais. Os dados serão analisados por meio de testes estatísticos, e sob a metodologia de análise temática. A diretriz de acolhimento contará com material baseado em diretrizes canadense e britânica.** Discussão** As diretrizes brasileiras de luto perinatal propostas são uma adaptação local das diretrizes canadense e britânica. Baseamo-nos na necessidade da família por memórias físicas e afetivas da criança morta para facilitar a vivência do processo do luto. Elas incluem os seguintes aspectos: (1) organização dos períodos da assistência a partir de suas respectivas necessidades, (2) criação do papel do Profissional do Luto, (3) ambientação das instituições, (4) disseminação das diretrizes e (5) criação de memórias do bebê. Espera-se que o projeto gere evidências adicionais para melhorar a saúde mental de mulheres e famílias que vivenciam uma perda perinatal.

**Registro do estudo**: RBR-3cpthr.

**Palavras-chave**: Óbito fetal, Óbito perinatal, Óbito neonatal, Luto gestacional, Luto perinatal, Luto neonatal, Luto materno, Luto parental, Protocolo de luto perinatal, Humanização da assistência ao parto.

## Supplementary Information

**Additional file 1.** Full Portuguese translation of The perinatal bereavement project: development and evaluation of supportive guidelines for families experiencing stillbirth and neonatal death in Southeast Brazil—a quasi-experimental before-and-after study.
