# Supplementary material for: The perinatal bereavement project: development and evaluation of supportive guidelines for families experiencing stillbirth and neonatal death in Southeast Brazil—a quasi-experimental before-and-after study
Source: Reprod Health. 2021 Jan 6;18:5. doi: 10.1186/s12978-020-01040-4 (PMC7787239; doi:10.1186/s12978-020-01040-4)
Supplement: Supplementary file 1 — Additional file 1. Full Portuguese translation of The perinatal bereavement project: development and evaluation of supportive guidelines for families experiencing stillbirth and neonatal death in Southeast Brazil—a quasi-experimental before-and-after study. [file 12978_2020_1040_MOESM1_ESM.docx]

**Projeto Luto Perinatal. Desenvolvimento e avaliação de diretrizes de acolhimento a famílias em processo de luto perinatal e neonatal no Sudeste do Brasil: um estudo quase-experimental do tipo antes e depois.**

Heloisa de Oliveira Salgado PhD^1^, Carla Betina Andreucci PhD^2^, Ana Clara Rezende Gomes^1^, João Paulo Souza PhD^1^.

1. Departamento de Medicina Social, Faculdade de Medicina de Ribeirão Preto, Universidade de São Paulo, Brasil

Endereço: Avenida dos Bandeirantes, 3900, Monte Alegre, Ribeirão Preto - SP - Brazil - 14049-900

1. Departamento de Medicina, Centro de Ciências Biológicas e da Saúde, Universidade Federal de São Carlos, Brasil

Endereço: Rod. Washington Luiz, s/n, São Carlos - SP, 13565-905

**Autor correspondente:**

**Heloisa de Oliveira Salgado**

**Afiliação**: Departamento de Medicina Social, Faculdade de Medicina de Ribeirão Preto, Universidade de São Paulo, Brasil

**Endereço**: Avenida dos Bandeirantes, 3900, Monte Alegre, Ribeirão Preto - SP - Brasil - 14049-900

**Telefone/fax**: +55 16 3315-3070

**E-mail**: [hellosalgado@gmail.com](mailto:hellosalgado@gmail.com)

# **RESUMO**

### **Contexto** Uma assistência avaliada positivamente por mães e pais que passaram pela perda perinatal permite a criação de memórias físicas e afetivas do bebê e possuem efeitos positivos no processo de luto da família. Este estudo avaliará os efeitos de uma diretriz de acolhimento na saúde mental de mulheres em processo de luto perinatal e neonatal em maternidades públicas do município de Ribeirão Preto (SP, Brasil). **Método** Estudo de métodos mistos (quantitativo e qualitativo), quase-experimental (antes e depois). A intervenção é a implementação de diretrizes de acolhimento ao luto de mulheres que tiveram um natimorto ou óbito neonatal. Um total de quarenta mulheres serão incluídas. Vinte participantes serão avaliadas antes, e vinte após a implementação da diretriz de acolhimento nas instituições. Serão aplicadas três escalas e uma entrevista semiestruturada para avaliar os efeitos da diretriz. Profissionais da saúde e gestores serão convidados a participar de grupos focais. Os dados serão analisados por meio de testes estatísticos, e sob a metodologia de análise temática. A diretriz de acolhimento contará com material baseado em diretrizes canadense e britânica. **Discussão** As diretrizes brasileiras de luto perinatal propostas são uma adaptação local das diretrizes canadense e britânica. Baseamo-nos na necessidade da família por memórias físicas e afetivas da criança morta para facilitar a vivência do processo do luto. Elas incluem os seguintes aspectos: (1) organização dos períodos da assistência a partir de suas respectivas necessidades, (2) criação do papel do Profissional do Luto, (3) ambientação das instituições, (4) disseminação das diretrizes e (5) criação de memórias do bebê. Espera-se que o projeto gere evidências adicionais para melhorar a saúde mental de mulheres e famílias que vivenciam uma perda perinatal.

### **Registro do estudo**: RBR-3cpthr

**Palavras-chave:** Óbito fetal, Óbito perinatal, Óbito neonatal, Luto gestacional, Luto perinatal, Luto neonatal, Luto materno, Luto parental, Protocolo de luto perinatal, Humanização da assistência ao parto.

**RESUMO EM LINGUAGEM LEIGA**

Para muitas pessoas, a gravidez não significa apenas gestar um bebê, mas ampliar a família com o nascimento de uma criança. Na maioria das vezes, as pessoas fazem planos e têm expectativas em relação a esse filho. Quando esses planos são interrompidos por causa de uma perda perinatal, a experiência se mostra traumática para a família.

Na cultura brasileira, validar o luto traumático pode ser muito difícil, especialmente quando a perda gestacional acontece muito cedo. Barreiras podem ser notadas não apenas pelo modo como a sociedade lida com o luto de mães e pais, mas também quando se observa o cuidado ofertado nas instituições de saúde às famílias enlutadas.

Criar memórias físicas e emocionais podem trazer satisfação a mães e pais em relação ao cuidado recebido quando um bebê morre. Essas memórias podem ser construídas quando há boa comunicação ao longo da prestação de cuidado; decisões compartilhadas; a chance de ver e segurar o bebê, assim como a chance de coletar memórias; ter privacidade e apoio contínuo durante todo o processo, incluindo quando há um novo período de gestação, parto e pós-parto. Com isso em mente, entre os fatores mais importantes estão a formação da equipe de saúde e de outros profissionais, a preparação da maternidade para apoiar famílias enlutadas e o apoio contínuo aos profissionais envolvidos no luto.

O artigo propõe diretrizes de acolhimento para apoiar as famílias que estão vivenciando um óbito fetal ou neonatal. Essas diretrizes podem ser utilizadas por profissionais que trabalham na assistência ao parto (enfermeiras obstétricas, obstetrizes, médicos obstetras, psicólogos e profissionais de saúde que trabalhem em maternidades), gestores, pesquisadores, formuladores de políticas e outras pessoas interessadas em desenvolver protocolos específicos para suas maternidades.

## **CONTEXTO**

A morte de um bebê durante a gestação, o parto ou no pós-parto é uma experiência traumática para a mulher e sua família [1,2]. Além disso, a experiência também pode ser traumática para os profissionais de saúde da assistência [2,3]. Muitos fatores tornam essa questão complexa e marginalizada, de modo que ela permanece oculta na agenda política: as taxas de natimortalidade não foram incluídas nos Objetivos de Desenvolvimento do Milênio, não foram monitoradas pelas Nações Unidas, tampouco incluídas nas medidas da Carga Global de Doenças [4], tornando-a um problema invisível. Ao mesmo tempo, abortos, óbitos fetais e neonatais levam ao luto parental, resultando em uma experiência complexa e, por vezes, traumática [5], uma questão desafiadora tanto para os profissionais de saúde quanto para as famílias. De acordo com Parkes (2009) [6], "Para a maioria das pessoas no mundo ocidental, a morte de uma criança é a mais aflitiva e dolorosa fonte de luto".

Adicionalmente, a crença de que a morte de um feto ou de um recém-nascido seria menos difícil de enfrentar em comparação com a morte de uma criança mais velha não é verdadeira [1]. A principal diferença entre essas duas situações é que a sociedade não reconhece as perdas perinatais; ao contrário, costuma-se minimizá-las, tornando-as invisíveis e silenciando a experiência de mães e pais. Esse tipo de luto é chamado de luto não reconhecido, compreendido como "[...] uma perda que não é ou não pode ser reconhecida abertamente, lamentada publicamente ou apoiada socialmente" (Doka, 1989) [7]. Portanto, mães e pais não têm seus sentimentos de luto, tristeza, vazio e impotência validados socialmente [8]. Por fim, mas não menos importante, mães, pais, famílias e profissionais de saúde não estão preparados para a interrupção imprevista de uma gravidez, ou a morte repentina de um bebê, o que torna a experiência ainda mais traumática e difícil de lidar [6].

Estima-se que houve 2,6 milhões de natimortos globalmente em 2016 [9]. Essas estimativas podem ser ainda mais altas, considerando-se a subnotificação de óbitos fetais e, se somarmos aos natimortos os óbitos neonatais [3,4], esses números podem ser ainda maiores. Eles indicam que a questão deve ser uma prioridade mundial; contudo, eles são apenas a ponta do iceberg.

Uma morte perinatal impacta a saúde mental de mães e pais, e pode desencadear sintomas depressivos, ansiedade, transtorno de estresse pós-traumático, ideação suicida, pânico e fobias [10]. Também há consequências para a esfera socioeconômica, com crises familiares, dificuldades ocupacionais e problemas relacionados com os altos custos dos cuidados em saúde [3].

As mulheres que dão à luz um bebê morto podem se sentir culpadas, e questionar sua competência para gerar uma criança saudável. Além disso, o processo de luto pode durar meses e anos e ainda impactar gestações subsequentes [10,11].

Dada a importância da saúde física e mental das mulheres que estão passando por esse processo, os profissionais de saúde devem considerar os sentimentos delas, ajudando-as a lidar com a culpa e aliviar as emoções negativas [12]. De forma semelhante, deve-se considerar que o cuidado de mães e pais enlutados traz estresse adicional para a equipe de saúde [5]. É difícil para a equipe de saúde das maternidades lidar com resultados negativos, em especial quando não há protocolos institucionais específicos [13]. Para estar apto a oferecer uma assistência mais qualificada, o profissional também precisa de acolhimento [5], formação, sessões de esclarecimento (*debriefing*) e apoio profissional [2].

As diretrizes e regulações ligadas a perda gestacional, natimortalidade ou óbito de recém-nascidos não constituem uma novidade. No Reino Unido, a iniciativa Sands (*Stillbirth & Neonatal Death Charity*) – uma instituição beneficente^1^ que trabalha com famílias enlutadas e profissionais de saúde – publicou um documento [14] bastante completo sobre essa questão. Países como Canadá [15], Austrália e Nova Zelândia [16], entre outros, também têm diretrizes, e a França^2^ possui leis específicas. Esses documentos reconhecem a importância dos filhos que morreram, e asseguram que o cuidado às famílias enlutadas tenha efeitos permanentes [1].

Até o momento, a literatura sobre o tema no Brasil é escassa. Recentemente, foi publicado um livro orientando a conduta de profissionais de saúde, com base na diretriz canadense [17]. Um estudo sobre lutos complicados comparando mulheres brasileiras e canadenses que perderam seus bebês identificou que as canadenses enfrentam um processo de luto menos complicado do que as brasileiras, sugerindo que os grupos profissionais de apoio ao luto, uma cultura incipiente no Brasil, podem fazer a diferença [18].

Um estudo brasileiro recente deu luz à dor não reconhecida de pais que sofreram perdas perinatais [19], discutindo a perspectiva masculina. Outro estudo do mesmo grupo destacou a lactação no contexto da perda perinatal [20]. Esta é uma questão relevante no Brasil, uma vez que, se oferecida alguma opção para a apojadura, a supressão farmacológica da lactação geralmente é a única oferecida às mulheres. Discussões preliminares do Projeto Luto Perinatal levaram à publicação de matéria em jornal de ampla circulação nacional, incluindo numerosas narrativas de mães enlutadas que enfrentaram diversos obstáculos para doação de leite materno.^3^ Tanto a reportagem, como as narrativas do livro "Como Lidar: Luto Perinatal" [17], trouxeram à tona o entendimento das mulheres de que doar leite materno poderia ajudá-las a lidar com a perda de seus bebês.

Em 2000, no documento “*Managing Complications in Pregnancy and Childbirth: A guide for midwives and doctors*” ('Gestão de complicações na gestação e no parto: Guia para enfermeiras obstétricas, obstetrizes e médicos', reimpresso em 2007), a OMS apresentou princípios a serem considerados no cuidado às famílias quando um bebê morre, tais como evitar a sedação materna, encorajar a mulher a ver e segurar seu bebê, coletar recordações do bebê, entre outros [21].

Em 2019, foi lançada a ação “*Why we need to talk about losing a baby*” ('Por que precisamos falar sobre a perda de um bebê') [22], com o objetivo de dar visibilidade mundial à atenção às mulheres que vivenciaram abortos, óbitos fetais e neonatais, assim como de descrever a necessidade de melhores práticas, e treinamento de profissionais de saúde. A iniciativa tinha como base as experiências de mulheres com perdas perinatais, bem como dados epidemiológicos, e propunha o fim do "estigma e vergonha inaceitáveis que as mulheres enfrentam depois da perda de um bebê" [22], reivindicando empatia, respeito e apoio durante o cuidado. Foi o início de um diálogo muito relevante sobre diretrizes de acolhimento a famílias com natimortos ou óbitos neonatais.

O desenvolvimento de protocolos baseados em evidências pode impactar positivamente a saúde de mães e pais que sofreram uma perda perinatal. O documento deve embasar-se em evidências trazidas pelos hospitais e pelos profissionais de saúde, e deve focar na criação de memórias do bebê que não sobreviveu. Um estudo norte-americano ouviu 40 mulheres que sofreram um aborto entre 12 e 20 semanas e receberam apoio baseado em protocolos de mediação [23]. Depois das intervenções, elas sentiram diminuição de desespero, e relataram que se sentiram cuidadas e assistidas. Os resultados provavelmente aplicam-se às demais perdas gestacionais, independentemente do tempo de gravidez.

Quando um bebê morre ou um aborto acontece, mães e pais podem ter muitos sentimentos de perda, incluindo dos planos que haviam feito para a vida do bebê, e de sonhos e expectativas que alimentavam em relação ao crescimento da família. A morte também modifica as expectativas que nutriam a respeito da parentalidade [15]. Olhando o luto de mães e pais através da lente da teoria de Rando (1993) [24], “Os Seis ‘R’ do Processo de Luto”, podemos encontrar na terceira fase - "lembrando e revivendo o falecido e o relacionamento" - enormes dificuldades para mães e pais em experienciar o luto gestacional ou neonatal, devido às poucas ou mesmo ausentes memórias reais do bebê que morreu ou da gravidez interrompida [15]. Com isto em mente, a coleta de lembranças é um ponto chave dentro das diretrizes abrangentes de luto perinatal.

Adicionalmente, realizar rituais de despedida e enfrentar a dor de forma realista são aspectos fundamentais para lidar com a morte: "Ver e segurar um bebê vivo logo após o nascimento é uma resposta normal dos pais. Ver e segurar um bebê nascido morto também é uma resposta normal, e há muitas evidências mostrando que pode ser uma experiência significativa e valiosa" [25]. Uma revisão sistemática em 2015 analisou os resultados de saúde associados às mães e pais que podiam ver e segurar seus bebês natimortos. Os resultados revelaram que permitir que as famílias tenham contato físico com seus filhos é benéfico, o que se opõe a conceitos anteriores indicando que profissionais de saúde deveriam desencorajar tal comportamento [26].

Há evidências em estudos nacionais que corroboram a importância do contato com o bebê, mesmo após a morte. Mães e pais mencionaram o desejo de manter a criança por perto, e relataram que sua dor foi diminuída devido à experiência incomparável e fundamental [27]. Outra investigação nacional concluiu que rituais que incluíam nomear, ver e tocar o bebê, bem como realizar cerimônias de despedida, contribuíram para um luto saudável [28].

Para que os profissionais de saúde possam garantir assistência adequada a mães e pais enlutados, é necessário que recebam formação e sejam instrumentalizados emocional e tecnicamente [18]. Lidar com mulheres que sofrem com a perda de um filho pode representar um enorme desafio, considerando suas singularidades culturais e pessoais [29,30,31,32]. Notavelmente, cuidar da equipe permite a preservação da continuidade e qualidade das assistências futuras em situações de perda [31,32].

## **Pergunta de pesquisa**

As diretrizes de acolhimento a famílias em processo de luto perinatal e neonatal, desenvolvidas para auxiliar mães e pais que estão vivenciando uma perda perinatal ou neonatal em maternidades brasileiras, promovem suporte e apoio que possibilitam a experiência de um luto saudável para a mulher e sua família?

**Hipóteses subjacentes**

Esta investigação pressupõe que os protocolos institucionais locais desenvolvidos a partir de diretrizes de acolhimento ao luto perinatal podem oferecer uma melhor assistência nos cenários de perda gestacional e neonatal. Ela se baseia no pressuposto de que os cuidados físicos e emocionais, assim como os momentos passados com o bebê e a coleta de lembranças, aumentam as chances de uma experiência de luto saudável.

**Estrutura conceitual**

O documento “*Pregnancy loss and the death of a baby: Guidelines for professionals - 4th Edition*" [14], publicado pela SANDS, foi utilizado para delinear a estrutura conceitual do presente estudo. Os autores consideraram que os principais pré-requisitos para oferecer cuidados de apoio a mães e pais enlutados são tempo, treinamento e suporte. Boa comunicação, decisão compartilhada e cuidados individuais são elementos importantes para oferecer assistência de alta qualidade a mães e pais enlutados. Estes são os aspectos fundamentais para as diretrizes de acolhimento e, por esta razão, estão no topo da matriz teórica conceitual, de acordo com a Figura 1.


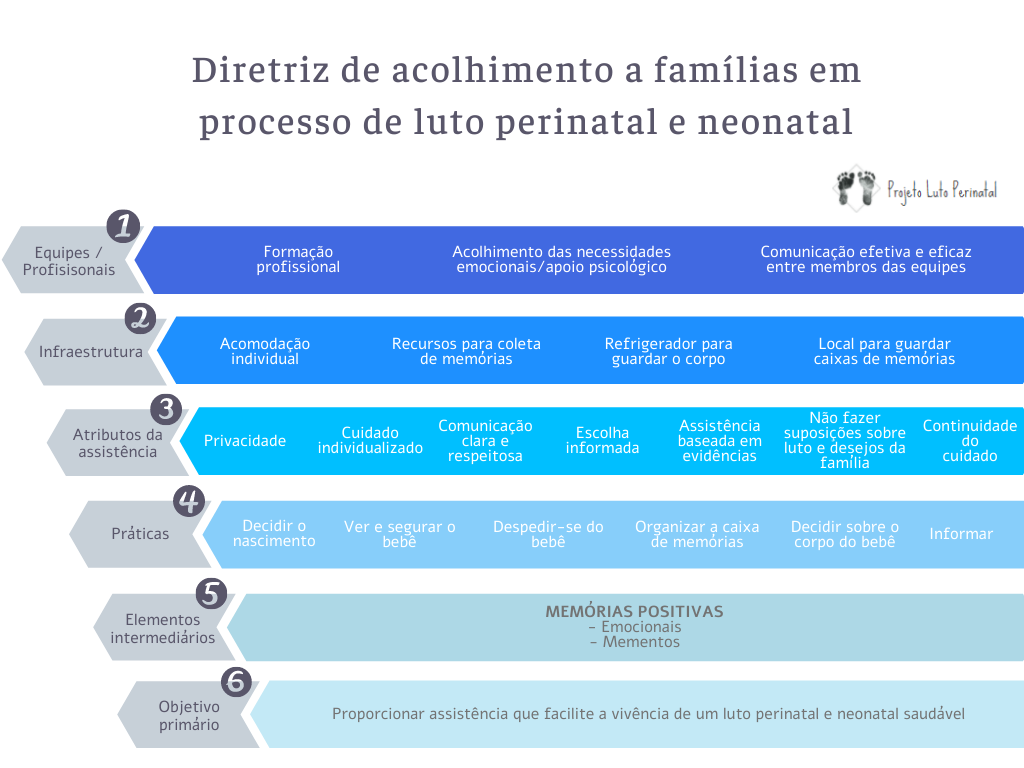


**Figura 1 – Diretriz de acolhimento a famílias em processo de luto perinatal e neonatal.**

Inicialmente, o primeiro nível da matriz contempla o trabalho com profissionais e equipes de saúde. Envolve três elementos, relativos à qualificação profissional, ao atendimento das necessidades emocionais dos profissionais (incluindo apoio psicológico) e à comunicação eficiente com a equipe. Considerando o cenário brasileiro, onde diretrizes e orientações são necessárias, o tripé a seguir é importante para delinear um ponto de partida para o planejamento de uma diretriz de acolhimento ao luto: (1) priorizar a formação inicial em relação às diretrizes e ao manejo da situação; oferecer formação contínua tanto aos funcionários qualificados quanto aos novatos, de modo que todos estejam alinhados com o modelo de atendimento a ser seguido; (2) compreender que os profissionais de saúde são, acima de tudo, humanos e, portanto, sujeitos à dor, à morte e ao luto em suas vidas profissionais e privadas. Eles também podem ser afetados por uma situação de luto ao mesmo tempo em que prestam assistência aos pacientes. Consequentemente, eles precisam de espaço institucional para atender às suas próprias necessidades emocionais. Com isto em mente, o Profissional do Luto é alocado para proporcionar cuidado e escuta sensível, sem qualquer julgamento. Esta ação visa atender às necessidades dos profissionais e da equipe; (3) oferecer boa comunicação, entre os profissionais e funcionários que também proporcionarão boa comunicação às famílias. Este é o aspecto central para garantir que os mães e pais estejam recebendo aquilo de que necessitam durante o processo de luto. A boa comunicação consiste em estabelecer e seguir protocolos com os quais toda a equipe de saúde deve estar alinhada.

O segundo nível da matriz está relacionado com a infraestrutura do estabelecimento de saúde que fornecerá à família memórias físicas e emocionais do bebê. Alguns dos itens da infraestrutura consistem na acomodação individual da família, recursos para coletar memórias, um refrigerador para preservar o cadáver e um local para guardar as caixas de memória. Os itens mencionados são fundamentais e a maioria das maternidades brasileiras precisa ser reestruturada de acordo com o seguinte: (1) a acomodação individual é uma das questões mais mencionadas por mães e pais brasileiros de luto. Ela os protege da curiosidade alheia; evita que eles tenham de compartilhar o momento de luto com outras famílias que estão celebrando a chegada de um bebê saudável ao seu lado; também lhes dá privacidade para conhecer seu filho momentos antes de enviá-lo aos rituais fúnebres. Mesmo com desafios logísticos nas maternidades brasileiras, alguma privacidade deve ser considerada e oferecida; (2) recursos para coletar lembranças dentro da maternidade, como uma câmera fotográfica para fotos do bebê e da família, ou material para registrar as impressões das mãos e dos pés, e uma mecha de cabelo do bebê, quando disponível. Uma caixa para guardar os itens é importante para o processo de criação de lembranças, e deve ser oferecida à família antes da alta hospitalar; (3) geladeira para preservar o cadáver é utilizada com menos frequência, mas quando necessária mostra-se essencial.

Em maternidades onde casos de alta complexidade são assistidos, muitas mulheres precisam de sedação ou ficam inconscientes (por período de tempo de dias ou mesmo semanas). Quando a mulher está acordada, após estar inconsciente durante o parto e nas primeiras horas ou dias após o parto, o impacto da notícia pode ser devastador, especialmente quando o bebê já foi enterrado ou cremado. Em tais situações, ter a chance de manter o corpo do bebê refrigerado para que a mãe possa se recuperar da sedação/inconsciência pode ter um resultado positivo para sua saúde mental; (4) ter um lugar para guardar as caixas de memória que não foram levadas pela família após a alta hospitalar também mostra-se relevante. É provável que estas famílias retornem nas semanas ou meses seguintes para levar as caixas, uma vez que decisões podem mudar com o passar do tempo.

Os atributos dos cuidados de saúde estão no terceiro nível da matriz. Eles destacam os aspectos essenciais do cuidado prestado a mães e pais: privacidade (conforme mencionado anteriormente) e cuidado individualizado, com adaptação da assistência às necessidades físicas, sociais e emocionais da família. Estes ajustes baseiam-se em alinhamentos de cuidados desenvolvidos previamente, no âmbito local. Este é um assunto delicado, e profissionais de saúde têm que observar valores éticos e sem julgamento sobre decisões, crenças e sentimentos das famílias. Trata-se de um desafio, pois profissionais são humanos, e podem ser individualmente afetados. Eles têm seus próprios mecanismos para lidar com a dor e isso tem que ser considerado, como discutido no primeiro nível da matriz. A sugestão para resolver este problema é o trabalho em equipe. Portanto, os profissionais devem ser apoiados pelo serviço de saúde e pelos protocolos estruturados, e podem seguir as recomendações preestabelecidas. Decisões informadas e conscientes, cuidados baseados em evidências, protocolos institucionais preestabelecidos e comportamento técnico uniforme são parte desta abordagem para produzir assistência segura e cuidado contínuo.

A práxis é descrita no quarto nível. Ela destaca a necessidade de decidir a via de nascimento como o primeiro passo. Consiste em discutir parto vaginal e cesárea, com a ressalva das proporções preocupantes de cesarianas no cenário brasileiro. Em alguns casos, quando a vida da mulher está em jogo, as cesáreas são realmente necessárias. No entanto, na maioria dos casos de natimortalidade, os partos vaginais são a escolha mais segura para a saúde da mulher.

A via de nascimento é uma decisão da mulher. No entanto, os profissionais de saúde devem explicar riscos e benefícios de cada opção. As mulheres devem ter a oportunidade de experimentar o trabalho de parto e o parto vaginal. Isso pode ajudá-las a iniciar o processo de luto, já que, com o tempo, as questões hormonais e físicas podem ser abordadas. Além disso, quando a mulher optar por um parto vaginal, ela participará ativamente do processo de parto biológico que culminará em ver e segurar seu bebê, assim como ter um momento de despedida, desde o trabalho de parto. Adicionalmente, ela provavelmente apresentará uma melhor condição física para participar dos rituais de despedida (funerais, cerimônias fúnebres ou cremação), e para engravidar novamente mais cedo, se desejar. Finalmente, as cesarianas aumentam os riscos em futuras gestações e partos, quando comparadas ao parto vaginal. Assim, todos os esforços da equipe devem envolver prestação dos melhores cuidados de saúde, evitando riscos físicos e psicológicos para a mãe. O parto vaginal tende a ser uma opção melhor sempre que possível, mas às vezes, especialmente quando há um trauma psicológico, uma cesárea deve ser considerada. Em qualquer caso, se for realizada uma cesárea, é recomendável encorajar a mãe a segurar o bebê. Isto posto, a equipe organizará a caixa de memória do bebê e orientará e apoiará no processo de tomada de decisão em relação ao corpo da criança. É também aconselhável, sempre que possível, ter planos escritos para o parto e pós-parto e para todas as decisões relacionadas com o bebê.

No Brasil, dificilmente se realizam exames *post mortem*. No entanto, sempre que possível, tais exames devem ser indicados pelos profissionais da saúde. Encontrar a causa da morte pode ajudar a família na elaboração do luto, embora um diagnóstico claro seja raro. Funerais, enterros ou cremações devem ser adiados para que exames necroscópicos possam ser realizados. Mesmo quando a morte ocorrer antes da viabilidade fetal (20 semanas e/ou 500g), a família poderá optar por funerais, de acordo com a legislação brasileira e os relatórios técnicos do Conselho Federal de Medicina [33]; é essencial que a família receba todas as informações que solicitou (por exemplo, procedimentos, perguntas clínicas), o que apoiará suas decisões.

Os elementos intermediários da matriz transcendem os aspectos objetivos aqui descritos. Eles incluem a criação de memórias positivas associadas com o bebê e sua morte, o que é desafiador e complexo. Para isso, recomenda-se personalizar a abordagem e respeitar as decisões da mulher, visando diminuir a ansiedade e as incertezas. Um protocolo institucional que seja uniforme e bem estruturado é essencial. Um diálogo aberto entre todas as pessoas envolvidas nos cuidados e no apoio emocional dado pelos profissionais são pontos fundamentais.

O final da matriz diz respeito ao objetivo principal desta proposta: oferecer cuidados que facilitem o luto perinatal saudável. Isto pode ser alcançado quando todas as medidas mencionadas anteriormente forem contempladas de forma transversal.

**Justificativa para o desenvolvimento de diretrizes de acolhimento à perda perinatal e neonatal no Brasil**

Até o momento, não há diretrizes de acolhimento à perda perinatal e neonatal no Brasil. Os serviços e os profissionais de saúde lidam com cada situação de acordo com suas próprias crenças e rituais institucionais. Às vezes o profissional oferece cuidados baseados no que lhe convém, já que lidar com pacientes em luto promove estresse e angústia. Além disso, não há apoio institucional disponível para ajudar a equipe hospitalar a cuidar de pacientes enlutados. A assistência médica frequentemente termina rapidamente, e o contato próximo é evitado, uma vez que lidar com famílias enlutadas é muitas vezes desafiador. Portanto, delinear diretrizes de acolhimento é urgente. Os serviços de saúde poderão criar protocolos locais para cuidar de famílias que vão experimentar a morte de um bebê. Da mesma forma, esses protocolos diminuirão o estresse dos profissionais de saúde.

As diretrizes de acolhimento devem ser usadas como referência para aqueles que desejam adaptá-las e implementá-las em diferentes contextos e cenários de cuidados de saúde, e utilizá-las localmente. Estas ferramentas podem promover a estrutura dos cuidados prestados às famílias que estão sofrendo perdas perinatais. Não obstante, o foco não é apenas o bem-estar da mulher grávida ou puérpera, mas também o bem-estar do profissional de saúde.

**OBJETIVOS**

O principal objetivo do Projeto Luto Perinatal é avaliar os efeitos das diretrizes internacionais de luto adaptadas ao contexto brasileiro na saúde mental e na experiência de luto de mães que sofreram uma perda perinatal. O objetivo secundário consiste em verificar a prevalência de depressão pós-parto, ansiedade, estresse e sintomas de adaptação ao luto, assim como avaliar os cuidados recebidos em dois momentos diferentes: antes e depois da capacitação de profissionais de saúde e adequação de maternidades.

**MÉTODOS**

**Desenho do Estudo**

Este é um estudo quase-experimental que usa métodos qualitativos e quantitativos. Também emprega uma abordagem analítica do tipo antes e depois.

**Local do Estudo**

***Procedimentos para a Coleta de Dados***

**Fases do Estudo:**

- - - 1. **Preparação**

Fase em que os encontros com as equipes das maternidades acontecerão, e os Profissionais de Referência de cada maternidade serão definidos. Os Profissionais de Referência serão responsáveis por: manter contato com os pesquisadores para solucionar dúvidas a respeito do projeto; organizar formações; informar a lista de casos de natimortos e de óbitos neonatais, entre outros. Eles também indicarão um Profissional do Luto para a maternidade. O Profissional do Luto é o responsável pelas questões do luto envolvendo famílias e equipe. Uma mesma pessoa pode acumular as funções Profissional de Referência e Profissional do Luto. Qualquer profissional que trabalha na maternidade, que tenha experiência significativa em suas rotinas e seus procedimentos e com a equipe, pode ser um Profissional de Referência. Pode haver duas pessoas responsáveis pela gestão e orientação e formação dos profissionais. Os Profissionais de Referência estarão em contato com a família quando o profissional de saúde alocado não estiver presente, ou quando ele pedir orientação. Podem ser médicos, enfermeiros ou obstetrizes.

O Profissional do Luto deve ter experiência e habilidade para oferecer apoio emocional e técnico à equipe. Deve estar disponível também para apoiar as famílias. Dessa maneira, os responsáveis pela maternidade devem decidir se há necessidade de um ou dois Profissionais do Luto. Idealmente, um psicólogo pode ser o Profissional do Luto, mas profissionais com mais habilidades podem assumir a posição. O Profissional do Luto receberá formação, com conteúdos teóricos e práticos, para que seja capacitado para prestar assistência, conforme requerido.

É o momento, então, de sensibilizar gestores, Profissionais de Referência e do Luto com relação aos processos de luto perinatal. Também é o momento de elaborar o Procedimento Operacional Padrão (POP), e realizar as formações.

- - - 1. **Pré-intervenção**

Esta fase consiste de duas ações: a) realização dos grupos focais e b) entrevistas com as mulheres.

1. *Grupo focal pré-intervenção*

Durante o grupo focal pré-intervenção, os participantes assinarão termo de consentimento para participação na pesquisa, e responderão questões sobre dados sociodemográficos e experiência de trabalho. O encontro terá duração de até 1h30.

1. *Entrevista pré-intervenção com as mulheres*

A entrevista pré-intervenção com as mulheres será realizada pelos pesquisadores, com base na lista de referência de natimorto e de óbito neonatal fornecida pelo Profissional de Referência das maternidades. Eles entrarão em contato para agendar uma entrevista face a face em local a ser definido. As informações relevantes para verificação dos critérios de inclusão e exclusão serão consideradas. As mulheres que atenderem aos critérios de inclusão receberão informações sobre o estudo, e serão convidadas a participar. Caso aceitem participar da pesquisa, a entrevista face a face será agendada. Durante o encontro, o termo de consentimento será assinado. Em seguida, as escalas de saúde mental serão aplicadas. A entrevista qualitativa compreenderá dados socioeconômicos, demográficos e comportamentais relacionados à perda pela qual as mulheres passaram.

- - - 1. **Intervenção**

A intervenção será baseada em duas publicações: o livro "Como Lidar: Luto Perinatal" [17], que por sua vez tem como fonte as “*Guidelines for health care professionals experiencing a perinatal loss”* [15], do Canadá, e o manual da Sands, “*Pregnancy loss and death of a baby: Guidelines for professionals”* [14]. Ambas as publicações trazem uma matriz para coordenar ações importantes na assistência às famílias (Figura 2).


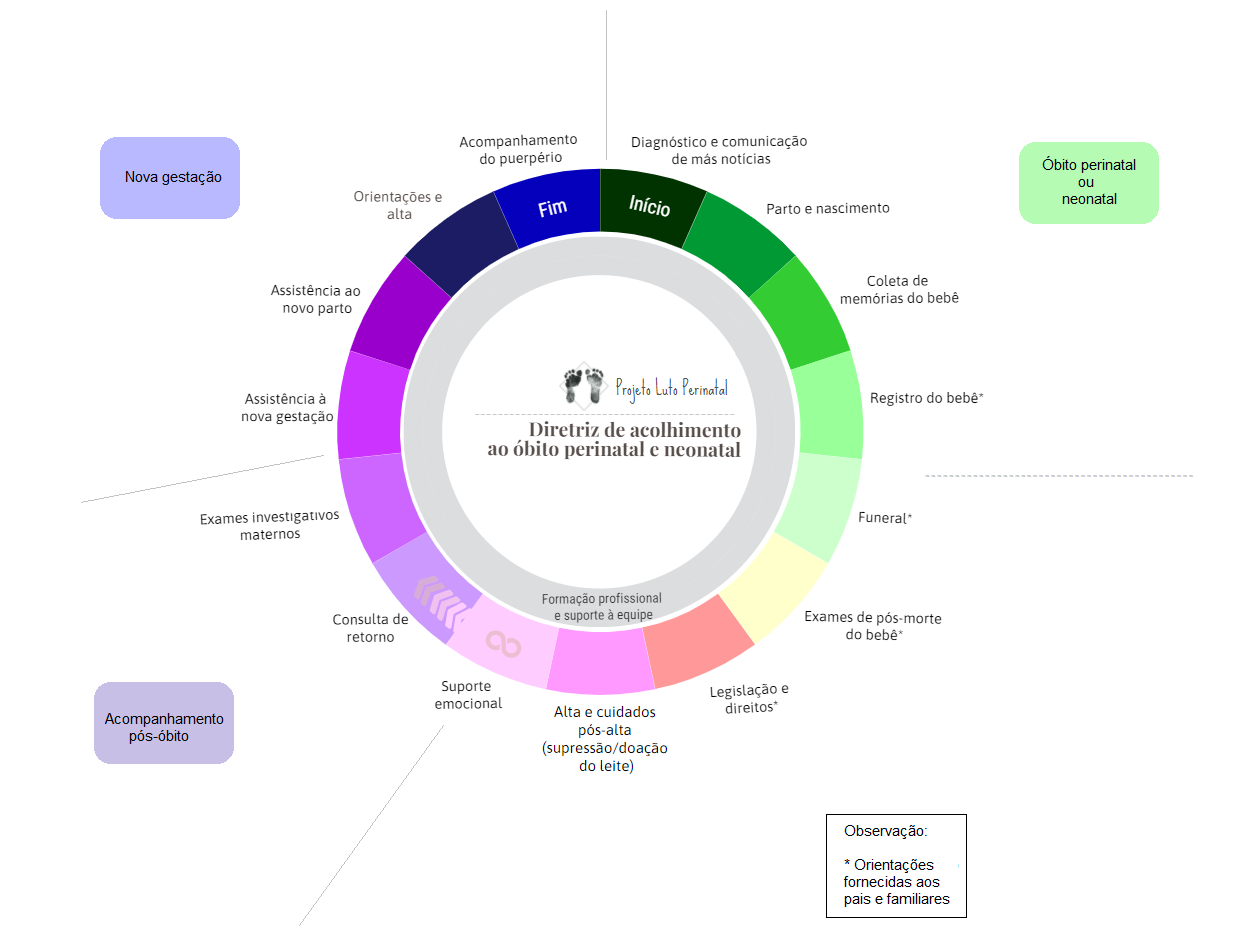


**Figura 2: Proposta de uma diretriz de acolhimento em casos de natimorto ou óbito neonatal.**

Materiais adicionais serão produzidos e publicados ao longo do processo de investigação. Com base no manual britânico já mencionado, o conteúdo inicial será ampliado. O material será disponibilizado no site [www.saude.fmrp.usp.br/lutoperinatal](http://www.saude.fmrp.usp.br/lutoperinatal). Todos os documentos necessários para a equipe hospitalar (arquivos sobre perda perinatal e criação de memória) também serão disponibilizados [nesse](http://www.saude.fmrp.usp.br/lutoperinatal) endereço. O arquivo sobre perda perinatal contém informações relevantes sobre saúde das mulheres, e o passo a passo das diretrizes. Ele ajudará os profissionais a compartilhar informações. O arquivo sobre criação de memórias orientará os passos relacionados à caixa de memórias do bebê. Um resumo das principais ações das diretrizes de acolhimento consta da Tabela 1.

[INSERT “TABELA 1” HERE]

Com relação ao modelo “Treinando quem Treina”, um professor especialista ensina um profissional que trabalha no estabelecimento de saúde. Quem recebe a formação ensina os demais. Assim, após a implementação do projeto, o profissional formado continuará a proporcionar formações para aqueles que precisarem, de forma matricial. Para este projeto, o especialista será um dos pesquisadores. Outro profissional participará do processo de qualificação, e ficará encarregado pela equipe regional após a finalização do projeto pelo especialista.

A metodologia de formação compreende os seguintes eventos:

- Evento sobre Luto Perinatal – Formação: um evento presencial que será organizado com o Departamento Regional de Saúde XIII (DRS-XIII) do estado de São Paulo. Apesar de ser focado em profissionais específicos, gestores e Profissionais de Referência, será aberto a todos os profissionais locais. Duração estimada: 3 horas.
- Encontro de Profissionais de Referência: este evento presencial tem como objetivo preparar os Profissionais de Referência e ajudá-los na prontidão em começar o projeto e escrever o POP. Eles também planejarão a qualificação dos profissionais de seus serviços. Participantes: profissionais indicados pelos gestores das maternidades (médicos, enfermeiros e psicólogos). Duração estimada: 3h.
- Encontro de Profissionais do Luto: um evento presencial com o objetivo de oferecer aos Profissionais do Luto formação teórica sobre luto, as perspectivas das famílias e dos profissionais, de modo que possam desempenhar suas funções. Participantes: profissionais indicados pelos gestores das maternidades (médicos, enfermeiros, psicólogos e assistentes sociais). Duração estimada: 4h.
- Encontro de sensibilização: um evento presencial em cada maternidade antes das formações das equipes. Tem como objetivo sensibilizar os profissionais da saúde e funcionários para o tema. Será produzido um vídeo para que outros profissionais do serviço que não participaram do encontro possam assistir.
- Rede de apoio para responder questões e orientar os profissionais envolvidos: durante toda a duração do projeto, os pesquisadores e o representante do DRS-XIII poderão ser contatados por telefone, via Whatsapp® (em grupo geral, grupos por maternidade e mensagens privadas).
- Haverá acompanhamento presencial e virtual até o fim do projeto.

Entre as ações, de acordo com a metodologia de formação, alguns itens serão entregues para as famílias. Adicionalmente, os seguintes itens serão entregues às equipes: uma câmera Polaroid® por instituição e kits contendo a uma caixa para manter as memórias, papel Polaroid®, cartão para impressão palmar e plantar, uma sacolinha plástica para a mecha de cabelo, uma sacola plástica maior para a roupinha do bebê, um cobertor que é usado quando o bebê é pequeno demais para as roupinhas convencionais.

- - - 1. **Pós-Intervenção**

Essa fase consiste em duas ações: entrevistas individuais com as mulheres do grupo pós-intervenção e grupos focais pós-intervenção. Um grupo focal compreende profissionais da enfermagem de cada maternidade e outro, os gestores. As entrevistas e os grupos focais espelharão os passos da fase pré-intervenção.

**Participantes do Estudo**

***Participantes***

Mulheres que tiveram um natimorto ou vivenciaram um óbito neonatal em uma das quatro maternidades públicas do município de Ribeirão Preto, Estado de São Paulo, Região Sudeste do Brasil. Também serão incluídos profissionais de saúde, trabalhadores e gestores das referidas maternidades.

1. *Participantes e critérios de elegibilidade*

Todas as quatro maternidades de Ribeirão Preto foram convidadas a participar do projeto e aceitaram.

Nenhum critério de exclusão foi estabelecido para profissionais de saúde, trabalhadores e gestores.

Os *critérios de inclusão* para as mulheres são:

1. residir em um dos municípios da região de Ribeirão Preto (Departamento Regional de Saúde XIII, Estado de São Paulo)
2. ter tido um bebê natimorto ou um óbito perinatal durante o período estimado para coleta de dados^^[[1]](#footnote-1)^^;
3. ter vivenciado o óbito do bebê em uma das circunstâncias a seguir:

- gestação que durou mais de 20 semanas completas;
- teve um bebê com pelo menos 500g;
- teve um bebê que faleceu em até 28 dias após o nascimento (óbito neonatal).

Os *critérios de exclusão* das mulheres são não compreender língua portuguesa e ter deficiência mental grave.

A principal diferença entre os grupos pré e pós-intervenção é o processo de formação que os profissionais de saúde receberão e a preparação da instituição para implementação do protocolo local. Supõe-se que as mulheres que tiverem bebê no grupo pré-intervenção não receberão cuidado baseado nas diretrizes de acolhimento ao luto perinatal.

As primeiras 20 mulheres de cada grupo que aceitarem participar do estudo serão entrevistadas.

*B) Instrumentos de pesquisa e fontes de informação*

Os dados serão produzidos por meio de entrevistas com as mulheres e dos grupos focais com profissionais. Será usado um questionário especialmente desenvolvido para o projeto, assim como ferramentas validadas usadas previamente em estudos sobre saúde mental e luto. Entrevistas-piloto serão conduzidas para validar o questionário e organizar o processo de entrevista.

Três escalas, adaptadas e validadas para o português do Brasil, serão aplicadas:

- *Escala de Luto Perinatal (ELP)*

A ELP apresenta 33 asserções psicométricas autoaplicáveis, divididas em três subescalas, relacionadas à sintomatologia da adaptação do luto perinatal. Envolve uma Escala de Likert de cinco pontos, de "discordo totalmente" a "concordo totalmente". O ponto de corte usado será 90, conforme validado por Paris et al. (2017) [34] para a população brasileira.

- *Escala de Depressão Pós-Parto de Edimburgo (EDPE)*

A EDPE é um conjunto de 10 questões de triagem autoaplicáveis, cada uma delas com quatro respostas de múltipla escolha, que indicam a intensidade dos sinais e sintomas depressivos presentes nos sete dias anteriores à aplicação. A pontuação de corte que sugere que a mulher provavelmente está deprimida (ou com sintomas de depressão) é ≥ 11, conforme validado por Santos [35] para a população brasileira.

- *Escala de Depressão, Ansiedade e Estresse* (EDAE-21)

A EDAE-21 é uma escala autoaplicável com sete itens cada. Ela avalia depressão, estresse e ansiedade, considerando a semana anterior à de sua aplicação. As respostas são dadas em uma Escala de Likert de quatro pontos, variando de 0 (discordo totalmente) a 3 (concordo totalmente). As variações de pontuação correspondem aos níveis de sintomas, como "normal" e "grave". As pontuações globais para os três construtos (depressão, estresse e ansiedade) serão calculadas conforme validado por Vignola et al. [36] para a população brasileira.

Um questionário sociodemográfico desenvolvido para coletar dados pessoais, informações comportamentais, histórico médico e informação sobre o cuidado recebido também será aplicado.

Cada maternidade indicará Profissionais de Referência e Profissionais do Luto a partir de seu quadro de colaboradores.

Os dados serão fornecidos pelos estabelecimentos de saúde. Eles enviarão uma lista de casos de natimortos e de óbitos neonatais, fornecida pelos respectivos Profissionais de Referência. Os pesquisadores entrarão em contato com as candidatas para verificar a elegibilidade para o estudo, convidando-as a participar e agendando um encontro.

Os grupos focais serão conduzidos pelos pesquisadores, e os profissionais de saúde participantes serão contatados pelo Profissional de Referência de cada maternidade.

**Resultados Primários e Secundários**

***Resultados Primários***

Um composto de um estado de luto (sintomas de adaptação do luto) e de taxas de depressão, estresse e ansiedade.

***Resultados Secundários***

- Absenteísmo no trabalho devido ao luto
- Tratamento psiquiátrico devido ao luto
- Internação materna em instituição psiquiátrica
- Tentativa de suicídio
- Suicídio

**Variáveis**

***Variáveis de Interesse***

[Insert “Tabela 2” here]

### **Viés**

### **Tabela 3: Questões possíveis e soluções**

|  | **Vieses Possíveis** | **Soluções Possíveis** |
| --- | --- | --- |
| 1 | Os profissionais de saúde podem desconsiderar orientações importantes ou podem ter dificuldade em mudar sua rotina no início. | O Profissional de Referência orientará os profissionais desde o início do projeto e ao longo de todo o processo. |
| 2 | Um erro em relação à idade gestacional ou o peso do bebê fornecido nos relatórios da maternidade pode incluir / excluir uma mulher da amostra. | As informações sobre o bebê (nascimento, óbito) serão verificadas com a mãe no primeiro contato. |
| 3 | Erros cometidos no preenchimento dos documentos gerados por este projeto. | O Profissional de Referência ajudará no preenchimento dos documentos e acompanhará as discussões dos casos. |
| 4 | A mãe / família não autoriza a coleta de alguns itens listados no projeto (foto, por exemplo). | O Profissional de Referência e o Profissional do Luto administrarão a situação e considerarão alternativas para resolver esta questão. Se necessário, os pesquisadores ajudarão a equacionar estas dificuldades. |
| 5 | Uma mulher do grupo pré-intervenção relata na entrevista que recebeu cuidados com base nas diretrizes de acolhimento em relação ao luto perinatal. | Durante o primeiro contato, serão solicitadas algumas informações que poderão ajudar a evitar esse viés. |

**Cálculo do Tamanho da Amostra ou Suficiência da Amostra Disponível**

Considerando os recursos financeiros e humanos do projeto, bem como a baixa prevalência dos eventos de interesse, optou-se por estabelecer o tamanho da amostra por conveniência (tamanho da amostra pragmática). Nesse contexto, o tempo para selecionar as mulheres que participarão foi fator decisivo para o tamanho da amostra. O tamanho da amostra em um estudo no mesmo campo foi de 44. Dada a quantidade de recursos disponíveis, fixamos o tempo pós-intervenção em seis meses. Assim, dado o tempo de seleção, o número estimado é de 40 mulheres (20 antes da intervenção e 20 após a intervenção).

**Aspectos Éticos e de Equidade**

Este estudo está em conformidade com a Declaração de Helsinque de 1975 [37], revisado em 2008, e segue as orientações e normas do Conselho Nacional de Saúde nº 466/12 [38]. Os dados coletados (entrevistas, escalas que foram traduzidas para o português brasileiro, escalas que foram validadas por investigações brasileiras) serão utilizados exclusivamente para pesquisas acadêmicas. A identidade e a privacidade dos participantes serão respeitadas.

Cada participante (mulheres que perderam seus bebês, profissionais e gestores) receberá o termo de consentimento. O termo de consentimento conterá informações sobre o estudo e telefone e e-mail de contato da equipe de pesquisa. O documento será lido e, em caso de concordância em participar da pesquisa, assinado. Uma das vias permanecerá com o pesquisador e, a outra, com o participante.

As mulheres que apresentarem algum risco à saúde mental serão encaminhadas para atendimento psicológico / psiquiátrico de acordo com a cidade de origem. Caso elas sejam diagnosticadas pela equipe do estabelecimento de saúde, serão seguidos os respectivos protocolos. Caso o diagnóstico seja conhecido na segunda entrevista, o pesquisador enviará os dados pessoais do participante (nome, aniversário, carteira de identidade, nome da mãe e cidade natal) ao Departamento Regional de Saúde XIII, aos cuidados do respectivo profissional de saúde. Este profissional tomará as providências cabíveis para que o caso seja devidamente acompanhado.

Este estudo não oferecerá aos participantes nenhum risco significativo. Os resultados serão aplicados para beneficiar a assistência a perdas perinatais em todo o país. Além disso, esta investigação visa promover a equidade, que pode ser alcançada quando são utilizados protocolos locais. Eles levarão em consideração os aspectos mais importantes das diretrizes de apoio, pois a assistência prestada é personalizada, respeitosa e digna. Dessa maneira, as necessidades físicas e emocionais da paciente serão atendidas.

**Gestão do Projeto**

A gestão do projeto será realizada pelos pesquisadores da Universidade de São Paulo, assim como pelo Comitê Condutor e pelos Comitês de Segurança e Monitoramento de Dados. A Assessoria Técnica será composta por uma pesquisadora da Universidade Federal de São Carlos e uma especialista do "Instituto de Psicologia 4 Estações"^4^, instituto que trabalha com o luto da cidade de São Paulo. O comitê de análise de dados será integrado por todos os pesquisadores.

**Gerenciamento e Análise de Dados**

## ***Plano de Análise de Dados***

Os dados serão analisados pela equipe do projeto e, sempre que necessário, por especialistas. O plano de análise, bem como um plano detalhado de análises estatísticas, será elaborado antes do início da coleta de dados. A análise temática qualitativa será realizada a partir dos dados produzidos pelos questionários, considerando categorias predeterminadas e novas categorias que a análise proporcionará. Os dados dos grupos focais serão tratados de maneira semelhante.

***Análise Descritiva***

A análise descritiva será baseada na análise temática qualitativa e na frequência de danos identificados pelo *Termômetro de Segurança do Luto Perinatal* desenvolvido para esta investigação (Figura 3).


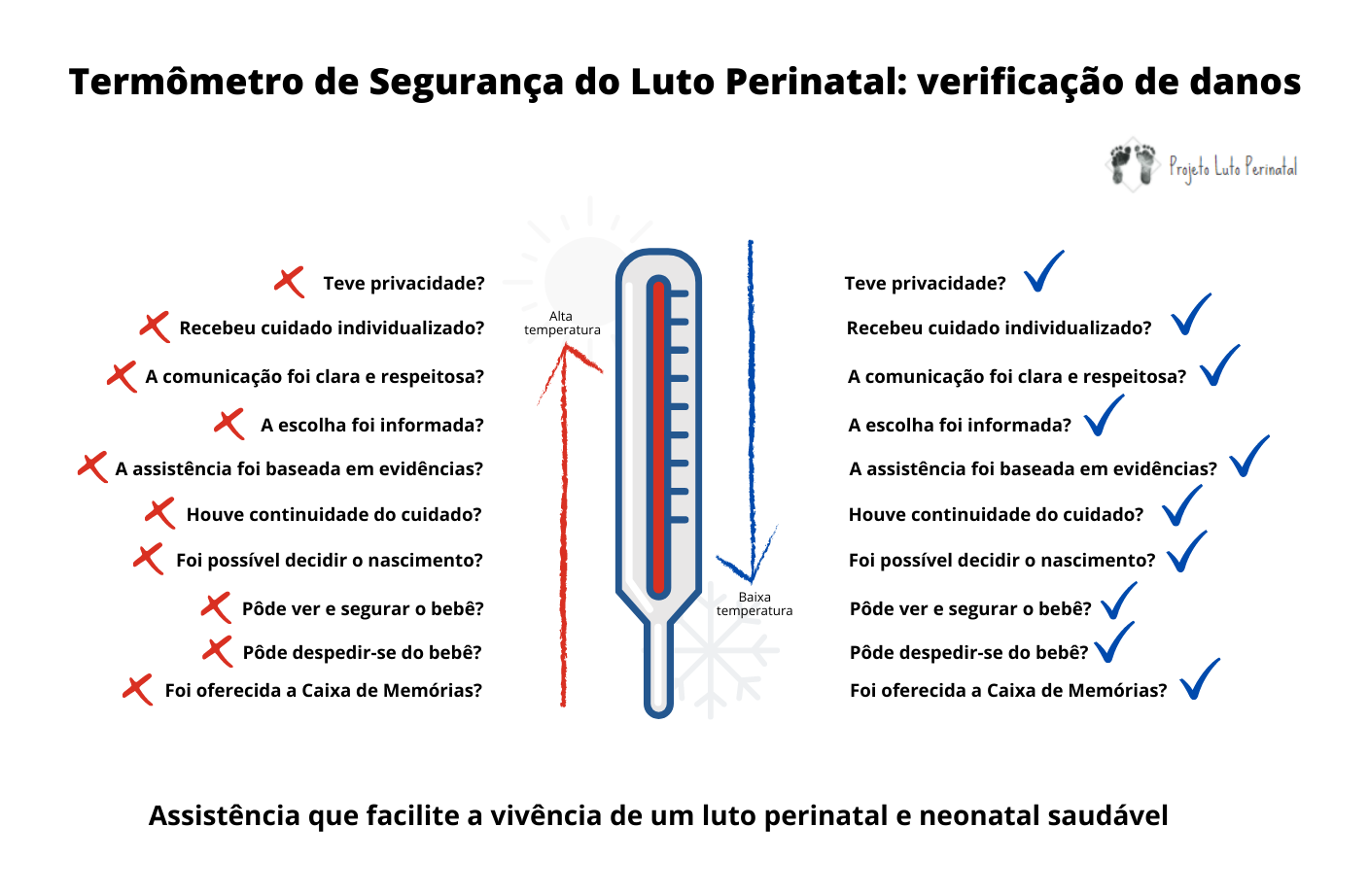


**Figura 3 – Termômetro de Segurança do Luto Perinatal: verificação de danos.**

O *termômetro de segurança do luto perinatal* fornece uma verificação de danos de 10 aspectos importantes que afetam a saúde mental materna e o processo de luto. É baseado nas ferramentas britânicas de *termômetros de segurança* desenvolvidas pelo “*National Health Service* (NHS)”, criadas para verificação de danos em cinco áreas diferentes [39].

De acordo com a ferramenta proposta para este estudo, baixas temperaturas de dano oferecem uma melhor chance de um luto perinatal saudável, uma vez que promovem melhores memórias para o processo de luto e a experiência de luto. Da mesma forma, partiu-se do pressuposto de que uma assistência ideal à saúde deve evitar situações que possam ser interpretadas como danos. Todos os dez itens previstos nesta ferramenta são considerados centrais e fazem parte das diretrizes britânicas [14] e/ou canadenses [15].

Será realizada análise descritiva para revelar as frequências de danos (Figura 3) ocasionados por atendimentos de saúde que não observaram as diretrizes de acolhimento para perdas perinatais. Esta análise também mostrará a relação entre esses danos e os resultados das três ferramentas usadas para verificar a saúde mental e o processo de luto das mulheres.

A análise temática também será realizada explorando os achados relacionados com os objetivos desta investigação. Serão comparados em cada grupo e entre os grupos. A análise considerará questões culturais e de gênero. Ela também contemplará especificidades institucionais que guiarão a implementação do protocolo em cada maternidade envolvida no projeto.

A análise temática qualitativa foi escolhida por permitir mapear, analisar e identificar padrões (tópicos) nos dados. Possui as seguintes etapas: 1) organização dos dados; 2) mapeamento de padrões, tópicos e categorias; 3) teste de hipóteses emergentes e 4) explicações alternativas obtidas dos grupos (pré-intervenção, pós-intervenção e grupo focal), bem como da análise comparativa entre os grupos.

***Análise Quantitativa***

Um plano detalhado para análise estatística será desenvolvido pelos pesquisadores antes do início da coleta de dados. Um plano de modelagem será desenvolvido e implementado por uma equipe de especialistas que inclui bioestatísticos, psicólogos e obstetras. Frequências simples serão calculadas e um método de comparação será usado (testes T-Student ou Qui-Quadrado).

### ***Publicação dos Achados e*** [***Plano de Disseminação dos Resultados***](http://scholar.google.com.br/scholar?q=results+dissemination+plan&hl=pt-BR&as_sdt=0&as_vis=1&oi=scholart)

Os achados serão publicados em periódicos científicos revisados por pares (em inglês ou em português do Brasil). Além disso, os resultados serão compartilhados localmente com os envolvidos no desenho do projeto, como forma de promover debates e reflexões sobre a prática corrente. Isso também ajudará a criar novas medidas para aprimorar os resultados no futuro, o que afetará as famílias passando por uma experiência de natimorto ou óbito neonatal.

# **DISCUSSÃO**

## ***Projeção dos Achados Principais***

A abordagem a mulheres e famílias que perderam seus bebês durante a gestação, o trabalho de parto ou pós-parto deve seguir uma diretriz de acolhimento específica, que deve ser adaptada localmente pelas maternidades brasileiras. A forma com que a questão é tratada por profissionais e estabelecimentos de saúde pode impactar o processo tanto positiva como negativamente. Quando as emoções negativas prevalecem, a abordagem técnica pode produzir efeitos psicológicos adversos duradouros, que podem permanecer por um longo período, algumas vezes por uma vida inteira.

O desenvolvimento das diretrizes brasileiras de acolhimento à perda perinatal, com base em experiências internacionais, pode preencher uma importante lacuna na saúde das mulheres. A proposta será contemplada a partir de uma perspectiva focada no desenvolvimento sustentável do bem-estar e do empoderamento das mulheres. Adicionalmente, as diretrizes incluem a qualificação profissional. A equipe que será envolvida nesse tipo de cuidado específico irá interagir para elaborar cenários situacionais e contextualizados para promover uma melhor relação entre profissionais de saúde e pacientes.

A qualificação do Profissional do Luto é outro aspecto relevante desta diretriz. O Profissional do Luto ajudará outros profissionais a lideram com questões específicas e complexas ligadas ao processo de luto, e ainda assistirá famílias enlutadas. Essa pessoa tratará assuntos privados e individuais da equipe e dos profissionais. Essas questões poderão surgir ao longo da assistência prestada às famílias enlutadas. Entre as funções do Profissional do Luto estão a reorganização da equipe, apoio psicológico para os envolvidos e escuta empática, sempre que necessário.

**Contribuições deste estudo para assistir natimortos e óbitos neonatais**

As diretrizes de acolhimento visam ajudar mulheres e famílias que estão vivenciando a natimortalidade e o óbito neonatal. Essa assistência é pautada pela empatia e eficiência neste momento desafiador. Além disso, propõe a continuidade do cuidado, incluindo confortar a mulher e sua família, auxiliar no parto e traçar procedimentos para o cuidado após a alta hospitalar. Isso pode contribuir para o bem-estar da mulher e, consequentemente, para sua qualidade de vida e sua saúde mental e física. De modo semelhante, a criação de procedimentos padronizados na instituição pode proporcionar mais satisfação para os profissionais, uma vez que o estresse ocupacional e a ansiedade na comunicação são reduzidos, e resultados obstétricos indesejados são mais bem administrados. As diretrizes oferecerão orientação prática em termos de rotinas técnicas relacionadas com a morte de um bebê durante a gestação. Serão incluídos itens como manejo do corpo e caixa de memórias, entre outras rotinas.

**Aplicabilidade Obstétrica**

A aplicabilidade dessas diretrizes resultará em um estudo piloto. Dessa forma, as diretrizes poderão ser replicadas em cenários semelhantes. Além disso, com base nestas diretrizes, outras maternidades ou organizações serão capazes de elaborar suas propostas para abordagem da perda perinatal. As instituições poderão usar os achados deste estudo para contemplar diferenças regionais em relação ao luto perinatal, como os de comunidades indígenas, quilombolas, favelas e outros grupos.

**Antecipando os Principais Problemas e Propondo Soluções**

Lidar com a morte em maternidades é uma situação delicada, podendo causar desconforto e quebra da privacidade entre pacientes e profissionais. Esses problemas poderão ser minimizados por meio de delineamento claro dos objetivos desta investigação. Serão destacados os benefícios que o projeto poderá trazer para famílias que estão passando por esse trauma, e para aquelas que poderão estar nessa situação no futuro.

Todos os participantes terão seu direito à privacidade preservado durante este projeto de pesquisa. Eles podem deixar o estudo a qualquer momento em que desejarem. Os autores / pesquisadores que vão lidar diretamente com os participantes estão preparados para ouvi-los com empatia e profissionalismo, de modo a evitar qualquer tipo de constrangimento e desconforto que esta investigação possa causar.

Por fim, qualquer problema ou dificuldade que o profissional possa ter em relação ao luto será atendido com gentileza, dentro do estabelecimento de saúde e, quando necessário, com psicólogo qualificado.

Algumas das limitações e dificuldades deste estudo podem ser verificadas na Tabela 4.

**Controle da Qualidade**

Os pesquisadores acompanharão a implantação dos protocolos locais. Isso será viável durante reuniões presenciais com os Profissionais de Referência e interações para promover a resolução de problemas no local. A qualificação dos profissionais e da equipe em relação aos protocolos será acompanhada pelos pesquisadores durante todo o projeto.

**Privacidade dos Dados**

A confidencialidade dos dados será protegida durante o projeto e, posteriormente, pelos pesquisadores e profissionais de TI envolvidos no grupo de pesquisa. O acesso ao conteúdo digitalizado será concedido por meio de senha criptografada e sistemas de segurança de dados. Será evitada a impressão de material e, caso isso seja necessário, será destruído após o projeto.

### **Sustentabilidade da Pesquisa**

***Estimativas de Impacto Ambiental***

O impacto ambiental da presente pesquisa será calculado pela ferramenta disponível em <https://www.tjpr.jus.br/web/gestao-ambiental/calculadoraco2> . O plano de compensação ambiental será elaborado com uma instituição local com sede em Ribeirão Preto que doa mudas e recursos para o plantio de árvores. Futuramente, com base na emissão de gás carbônico, as mudas serão plantadas de acordo com as instruções da Prefeitura de Ribeirão Preto. O número de árvores irá compensar as emissões de CO2 que aconteceram durante esta investigação.

O plantio de árvores acontecerá durante evento semelhante aos que já acontecem em outras cidades brasileiras – Araraquara (SP), Goiânia (GO), Recife (PE) –, onde mães e pais que tiveram óbitos gestacionais ou neonatais vão plantar uma árvore para homenagear o filho que perderam. Assim, um parque / jardim / canteiro de memórias será criado. Este evento deve ocorrer anualmente, para que mais mães e pais possam se juntar àqueles que já plantaram suas árvores. Isso tem um resultado positivo considerando não só a perspectiva do luto, mas também a ambiental.

**Lista de Abreviaturas**

EADS-21 – Escala de Depressão, Ansiedade e Estresse-21

ELP – Escala de Luto Perinatal

EDPE – Escala de Depressão Pós-Parto de Edimburgo

POP – Protocolo Operacional Padrão

OMS – Organização Mundial da Saúde

**DECLARAÇÃO**

***Aprovação Ética e Consentimento para Participar e Consentimento para Publicação***

Este estudo está em conformidade com a Declaração de Helsinque de 1975 [37], revisado em 2008, e segue as orientações e normas do Conselho Nacional de Saúde n^º^ 466/12 [38]. O estudo foi aprovado pelo comitê de ética do Hospital das Clínicas da Faculdade de Medicina de Ribeirão Preto da USP (HCFMRP/USP), Ribeirão Preto, Brasil, protocolo 3.275.502, em 22 de abril de 2019. Este estudo obterá consentimento de todos os participantes.

***Consentimento para Publicação***

O termo de consentimento dos participantes da pesquisa será armazenado e permanecerá disponível mediante solicitação.

***Disponibilidade dos Dados e Materiais***

Os conjuntos de dados usados e / ou analisados durante o presente estudo estão incluídos neste artigo publicado e informações suplementares podem ser acessadas mediante solicitação razoável.

***Conflitos de Interesses***

Todos os autores declaram que não têm conflitos de interesses.

***Financiamento***

A execução do projeto será financiada pela bolsa anual de pesquisa da Fundação de Apoio ao Ensino, Pesquisa e Assistência (FAEPA) do Hospital das Clínicas da Faculdade de Medicina de Ribeirão Preto da Universidade de São Paulo (HCFMRP-USP).

***Contribuições dos Autores***

HOS e JPS desenvolveram o estudo e escreveram o protocolo. ACR e CBA discutiram o manuscrito e adicionaram suas contribuições ao texto. Todos os autores leram e aprovaram a versão final.

***Agradecimentos***

Agradecemos a Dra. Valéria Tinoco, do "Instituto de Psicologia 4 Estações", psicóloga especialista em luto, por sua leitura e contribuição para o texto, e Beatriz de Oliveira, linguista e tradutora, pela edição e revisão do manuscrito em inglês.

***Informações sobre os Autores***

Departamento de Medicina Social. Faculdade de Medicina de Ribeirão Preto. Universidade de São Paulo. Ribeirão Preto, São Paulo, Brasil. Heloisa de Oliveira Salgado, Ana Clara Rezende Gomes e João Paulo Souza.

Centro de Ciências Biológicas e da Saúde. Departamento de Medicina. Universidade Federal de São Carlos (UFSCar). São Paulo, Brasil. Carla Betina Andreucci.

***Notas de rodapé***

1. SANDS (Stillbirth & Neonatal Death Charity): <https://www.sands.org.uk/>.
2. Circulaire interministérielle DGCL/DACS/DHOS/DGS/DGS/2009/182 du 19 juin 2009: <http://web.archive.org/web/20130226185927/http://www.sante.gouv.fr/IMG/pdf/circulaire_182_190609.pdf>.
3. Mulheres que perderam bebês em fase final da gestação querem opção de doar leite materno: <https://www1.folha.uol.com.br/cotidiano/2019/10/mulheres-que-perderam-bebes-em-fase-final-da-gestacao-querem-opcao-de-doar-leite-materno.shtml>.
4. Instituto de Psicologia 4 Estações: <https://www.4estacoes.com/>.

**REFERÊNCIAS BIBLIOGRÁFICAS**

[1] Scott J. Stillbirths: breaking the silence of a hidden grief. Lancet. 2011;377:1386-8.

[2] Homer CSE, Malata A, Ten Hoope-Bender P. Supporting women, families, and care providers after stillbirths. Lancet. 2010; 387:516-517.

[3] Heazell AEP, Siassakos D, Blencowe H, Burden C, Bhutta ZA et al. Stillbirths: economic and psychosocial consequences. Lancet. 2016;387:604-616.

[4] Lawn JE, Blencowe H, Pattinson R, Cousens S, Kumar R et al. Stillbirths: Where? When? Why? How to make the data count? Lancet. 2011;377:1448–63.

[5] Farrales LL, Cacciatore J, Jonas-Simpson C, Dharamsi S, Ascher J, Klein MC. What bereaved parents want health care providers to know when their babies are stillborn: a community-based participatory study. BMC Psychol. 2020;8:18.

[6] Parkes CM. Love and Loss: The Roots of Grief and its Complications. London: Routledge.

[7] Doka K. Disenfranchised Grief: Recognizing Hidden Sorrow. New York: Lexington Books;1989.p.04.

[8] Casellato G. Luto não reconhecido: um conceito a ser explorado. In: Casellato G, editor. Dor silenciosa ou dor silenciada? Perdas e lutos não reconhecidos por enlutados e sociedade. Campinas: Livro Pleno; 2005. p.19-33.

[9] Lawn JE, Blencowe H, Waiswa P, Amouzou A, Mathers C et al. Stillbirths: rates, risk factors, and acceleration towards 2030. Lancet. 2016;387:587–603.

[10] Hutti MH. Social and Professional Support Needs of Families After Perinatal Loss. J Obstet Gynecol Neonatal Nurs. 2005;34:630-638.

[11] Armstrong DS. Impact of Prior Perinatal Loss on Subsequent Pregnancies. J Obstet Gynecol Neonatal Nurs. 2004; 33:765-73.

[12] Murphy S. Reclaiming a moral identity: stillbirth, stigma and ‘moral mothers’. Midwifery. 2012;28:476-480.

[13] Setubal MSV, Antonio MARGM, Amaral EM, Boulet J. Improving Perinatology Residents' Skills in Breaking Bad News: A Randomized Intervention Study. Rev Bras Ginecol Obstet. 2018;40:137-146.

[14] Schott J, Henley A, Kohner N. Pregnancy loss and the death of a baby: guidelines for professionals. 4thEd. UK: Tantamount on behalf of Sands, the Stillbirth & Neonatal Death Charity; 2016.

[15] Canadian Pedriatic Society Statement. Guidelines for health care professionals supporting families experiencing a perinatal loss. Paediatrics & Child Health. 2001;6(7):469-477.

[16] Perinatal Society of Australia and New Zealand Clinical Practice Guideline for Care Around Stillbirth and Neonatal Death, Third Edition, June 2018. <https://www.stillbirthcre.org.au/assets/Uploads/Respectful-and-Supportive-Perinatal-Bereavement-Care.pdf> Accessed 02 Sep 2020.

[17] Salgado HO, Polido CA. Como Lidar: Luto Perinatal: Acolhimento em situações de perda gestacional e neonatal.1ed. São Paulo: Ema Livros; 2018.

[18] Paris GF, Montigny F, Pelloso SM. Fatores associados ao estado de luto após óbito fetal: estudo comparativo entre brasileiras e canadenses. Rev. Esc. Enferm. 2016;50:546-553.

[19] Galvão GMM, Morsch DS, Tavares EC, Bouzada MCF, Byrd STE. An Unrecognizable Pain: Neonatal Loss and The Needs of Fathers. American International Journal of Humanities, Arts and Social Sciences. 2020;2(3).

[20] Galvão GMM, Tavares EC, Costa EA, Tavares GR, Bouzada MCF, Byrd SE. A Mother’s Account About Lactation in the Context of Perinatal Death. American International Journal of Humanities, Arts and Social Sciences. 2020;2(3).

[21] WHO, UNICEF, UNFPA. Managing complications in pregnancy and childbirth: a guide for midwives and doctors. 2nd ed. Geneva: World Health Organization; 2017.

[22] WHO. Why we need to talk about losing a baby. Geneva: World Health Organization; 2019. <https://www.who.int/news-room/spotlight/why-we-need-to-talk-about-losing-a-baby> . Accessed 02 Sep 2020.

[23] Johnson OP, Langford RW. A Randomized Trial of a Bereavement Intervention for Pregnancy Loss. J Obstet Gynecol Neonatal Nurs. 2015; 44:492-499.

[24] Rando TA. A perspective on loss, grief and mourning. In: Rando TA, editor. Treatment of Complicated Mourning. Champaign: Research Press;1993. p.19-77.

[25] Limbo R. Caring for Families Experiencing Stillbirth: A Unified Position Statement on Contact with the Baby. Illness, Crisis & Loss. 2012;20:295-298.

[26] Kingdon C, Givens JL, O'Donnell E, Turner M. Seeing and Holding Baby: Systematic Review of Clinical Management and Parental Outcomes After Stillbirth. Birth. 2015;42:206-218.

[27] Silva JDD, Sales CA. Do imaginário ao real: a experiência de pais enlutados. Rev Rene. 2012; 13: 1142-51.

[28] Oishi KL. O jardim de Julia: a vivência de uma mãe durante o luto. Psic.: Teor. e Pesq. 2014;30:5-11.

[29] Thomas J. The Child Bereavement Trust: caring for bereaved families. British Journal of Midwifery. 1997;5:474–477.

[30] Montero SMP, Sánchez JMR, Montoro CH, Crespo ML, Jaén AGV, Tirado MBR. Experiences with perinatal loss from the health professionals’ perspective. Rev Lat Am Enfermagem. 2011;19:1405–1412.

[31] Flenady V, Boyle F, Koopmans L, Wilson T, Stones W, Cacciatore J. Meeting the needs of parents after a stillbirth or neonatal death. BJOG. 2014;121:137–140.

[32] Homer CSE, Malata A, Hoope-Bender PT. Supporting women, families, and care providers after stillbirths. Lancet. 2016;387:516–517.

[33] Ministério da Saúde. Agência Nacional de Vigilância Sanitária. Resolução RDC Nº 306. 2004. <http://bvsms.saude.gov.br/bvs/saudelegis/anvisa/2004/res0306_07_12_2004.html> . Accessed 17 Aug 2020.

[34] Paris GF, Montigny F, Pelloso SM. Cross-cultural adaptation and validation evidence of the perinatal grief scale. Texto contexto - Enferm. 2017;26(1): e5430015.

[35] Santos IS, Matijasevich A, Tavares BF, Barros AJD, Botelho IP, Lapolli C et al. Validation of the Edinburgh Postnatal Depression Scale (EPDS) in a sample of mothers from the 2004 Pelotas Birth Cohort Study. Cad. Saúde Pública. 2007;23(11):2577-2588.

[36] Vignola RCB, Tucci AM. Adaptation and validation of the depression, anxiety and stress scale (DASS) to Brazilian Portuguese. Journal of Affective Disorders. 2013;155:104–109.

[37] World Medical Association. WMA Declaration of Helsinki - Ethical Principles for Medical Research Involving Human Subjects. <https://www.wma.net/policies-post/wma-declaration-of-helsinki-ethical-principles-for-medical-research-involving-human-subjects/>. Accessed 02 Sep 2020.

[38] Conselho Nacional de Saúde. Resolução nº 466, de 12 de dezembro de 2012. http://conselho.saude.gov.br/resolucoes/2012/Reso466.pdf. Accessed 02 Sep 2020.

[39] NHS. Safety Thermometer. <https://www.safetythermometer.nhs.uk/>. Accessed 02 Sep 2020.

**FIGURAS, TABELAS E ARQUIVOS ADICIONAIS**

**Tabela 1 – Resumo das principais ações das diretrizes de acolhimento a famílias vivendo uma situação de óbito perinatal e neonatal.**

| **Resumo das diretrizes de acolhimento sobre luto perinatal** | |
| --- | --- |
| **O que fazer quando há:**   - ***Natimorto*** - ***Óbito neonatal ou iminência de óbito neonatal*** - ***Malformação grave (incompatível com a vida ou com alta probabilidade de óbito)*** | 1. Assegure a privacidade da mulher / família; 2. Faça a comunicação de má notícia adequadamente; 3. Dê à família toda a informação necessária e solicitada; 4. Estimule a preparação de um Plano de Cuidado para a mulher e o bebê. O Plano de Cuidado inclui um plano de parto, sempre que possível, e cuidados paliativos para o bebê, sempre que necessário; 5. Selecione e prepare um quarto privativo, para evitar contato com outras mães e seus bebês; 6. Cole adesivos especiais no quadro de avisos e na porta do quarto da mãe enlutada, de modo que a equipe saiba de sua condição; 7. Evite a sedação; 8. Respeite os planos iniciais – sempre que possível; 9. Estimule a mãe / família a ver o bebê; 10. Estimule a mãe / família a tocar o bebê, e permanecer um tempo com a criança; 11. Providencie a caixa de memórias. |
| **Como preparar a família para ver o bebê:** | - Limpe o bebê (não dê banho); - Vista o bebê com fraldas, meias, touca, roupas, cobertor, etc.; - Siga o mesmo protocolo da maternidade usado para levar bebês vivos para o colo de suas mães; - Chame o bebê pelo nome; - Acompanhe a mãe / família, mas mantenha certa distância. Nunca as deixe sem assistência. |
| **O que deve ser providenciado para a caixa de memórias:** | - Mecha de cabelo; - Impressão palmar e plantar; - Impressão da placenta (opcional); - Fotos do bebê; - As primeiras roupas do bebê; - Pulseira de identificação do bebê; - Cartões e cartas escritos pela equipe; - Folhetos sobre o processo de luto e grupos de apoio ao luto. |
| **Que tipo de orientação escrita deve ser fornecida:** | - Todas as informações sobre serviços funerários e cartório de registro civil; - Informação sobre como lidar com a produção de leite materno;   - Supressão da lactação;   - Doação de leite materno; - Cuidado pós-alta; - Consultas médicas e exames adicionais para investigações aprofundadas (se necessário); - Grupo de apoio ao luto; - Serviços psicológicos. |
| **O que fazer para proporcionar cuidado contínuo:** | - Discuta planejamento reprodutivo; - Agende consultas regulares para avaliar a saúde física e mental, discuta novos achados sobre o que aconteceu com o bebê (quando aplicável); - No caso de uma nova gestação, siga as diretrizes de saúde para gestantes com experiências prévias de perda de um bebê; - Providencie assistência à mulher durante o novo puerpério. |
| **Diretrizes para assistência a gestantes com experiências prévias de perda de um bebê:** | - Lembre-se: esta não é uma gestação típica e, do ponto de vista da saúde mental, pode ser considerada uma gestação de risco; - A perda prévia pode impactar a gestação seguinte da mãe enlutada, do pré-natal até o puerpério e a amamentação; - Medo e queixas são respostas esperadas e devem ser reconhecidos; - Algumas mães e pais preferem receber cuidados em uma maternidade diferente ou por profissionais diferentes da equipe, o que não deve ser um problema; - Rastreio em saúde mental deve estar disponível para mães e pais; - Apoio especializado em saúde mental ou consultas de psicologia devem ser estimulados a qualquer momento durante a gravidez e o pós-parto; - Considere evitar educação perinatal padrão, priorizando sessões de preparação individual para o trabalho de parto, parto e cuidados com o recém-nascido; - Um adesivo especial pode ser usado na placa e na porta do quarto quando houver uma mãe que já sofreu previamente uma perda perinatal, de forma que a equipe saiba de sua condição; - Estimule a elaboração de um plano de parto, de modo que as preferências possam ser compartilhadas com facilidade com a equipe durante o trabalho de parto, o parto e o pós-parto. - Considere agendar consultas de pré-natal extras e mais longas. Opções extras de rastreamento devem ser consideradas sempre que necessário ou demandado; - Reserve um dia/horário semanal em sua instituição para que gestantes que passaram previamente por uma perda perinatal busquem atendimento caso estejam em dúvida sobre o bem-estar do seu bebê e da evolução sem complicações da gestação; - Uma cesárea sem indicação clínica pode ser considerada, quando o medo do parto natural não foi resolvido após várias intervenções psicológicas e de comunicação, desde que as mulheres reconheçam os riscos associados. |

**Tabela 2 – Variáveis de Interesse.**

|  | **Variáveis de**  **Interesse** | **Fonte de dados** | **Métodos estatísticos** |
| --- | --- | --- | --- |
| **Dados**  **Sociodemográficos:** | - Idade; - Estado conjugal; - Escolaridade; - Condição socioeconômica; - Cor da pele; - Emprego; - Religião. | - Questionário. | - Análise descritiva. - Análise de frequência. |
| **Características / Informações pessoais e sobre comportamentos** | - Faz atividades recreativas (atividades físicas e artísticas); - Pratica esportes; - Usa drogas (álcool, cigarro e/ou drogas ilícitas); - Está sob medicação e/ou tratamento psiquiátrico; - Participa de grupos religiosos; - Faz psicoterapia; - Participa de grupos de apoio ao luto. | - Questionário. | - Análise descritiva. - Análise de frequência. |
| **Histórico de saúde** | - Idade gestacional; - Abortos, natimortos ou óbitos neonatais anteriores; - Doenças prévias à gestação; - Doenças nesta gestação; - Malformação fetal; - Complicações no parto. | - Questionário. | - Análise descritiva. - Análise de frequência. |
| **Dados de avaliação da intervenção** | - Satisfação com a assistência prestada. | - Questionário. | - Análise descritiva. Análise de frequência. |
|  | - Nível de percepção sobre ansiedade, estresse, depressão e depressão pós-parto. | - Escala de Depressão Pós-Parto de Edimburgo. - Escala de Depressão, Ansiedade e Estresse-21. | - Análise descritiva. - Análise de frequência. |
|  | - Nível de percepção sobre sinais (sintomas e sentimentos) de luto. | - Escala de Luto Perinatal. | - Análise descritiva. - Análise de frequência. |
|  | - Dificuldades de retomar rotina da vida. | - Escala de Luto Perinatal. | - Análise descritiva. - Análise de frequência. |

**Tabela 4: Antecipando Dificuldades: Estratégias de Mitigação.**

|  | **Dificuldades** | **Estratégias de mitigação** |
| --- | --- | --- |
| 1 | Os participantes podem exibir emoções fortes, como tristeza, desespero, raiva, etc. | Os entrevistadores estarão preparados para essas situações e irão interromper e reagendar a entrevista, se necessário. |
| 2 | Os participantes podem sentir-se emocionalmente incomodados em compartilhar suas experiências e sentimentos em relação à perda do bebê, uma vez que estão de luto. | O questionário é muito detalhado e as informações fundamentais para entender o atendimento são baseadas em perguntas de sim / não. |
| 3 | Os profissionais de saúde podem estar passando por um momento de luto em suas vidas. Além disso, situações de luto podem desencadear sentimentos anteriores relacionados a perdas ocorridas em sua vida profissional ou privada. Isso pode comprometer ou limitar a assistência. | O Profissional do Luto prestará muita atenção a este problema e poderá apoiar o profissional e / ou reorganizar a equipe e substituir este membro. |
| 4 | A maternidade pode não ter recursos para seguir as novas diretrizes para luto perinatal. | Recursos limitados são tratados por este projeto. É possível ajustá-los de acordo. |
| 5 | Profissionais e demais trabalhadores podem sentir algum desconforto ou achar as orientações mórbidas. | O Profissional de Referência ajudará os profissionais e a equipe a se adaptarem à nova rotina. Profissionais mais adaptados podem se juntar à equipe para ajudar na transição. |
| 6 | Dificuldades no cuidado contínuo após a alta hospitalar. | O representante da Diretoria Regional de Saúde XIII (Saúde da Mulher) acompanhará a implementação do projeto e abordará esse problema. |
| 7 | A maternidade não quer compartilhar dados (como telefone da mulher, e-mail, etc.). | A equipe de gerenciamento do projeto entende que, caso a maternidade não queira compartilhar dados, ela pode ser incluída no treinamento da equipe, mas não na coleta de dados. O treinamento e a coleta de dados não são processos imbricados. |

1. Devido à pandemia de Covid-19, o período de recrutamento das mulheres será modificado. [↑](#footnote-ref-1)
